# Stroke care during the COVID-19 pandemic: Case numbers, treatments, and mortality in two large German stroke registries

**DOI:** 10.3389/fneur.2022.924271

**Published:** 2022-07-22

**Authors:** Alicia Brunssen, Viktoria Rücker, Peter Heuschmann, Jana Held, Peter Hermanek, Ansgar Berlis, Martin Hecht, Klaus Berger

**Affiliations:** ^1^Institute of Epidemiology and Social Medicine, University of Münster, Münster, Germany; ^2^Institute of Clinical Epidemiology and Biometry, University of Würzburg, Würzburg, Germany; ^3^Clinical Trial Center (P.U.H.), Würzburg University Hospital, Würzburg, Germany; ^4^Bavarian Working Party for Quality Assurance, Munich, Germany; ^5^Department of Diagnostic and Interventional Neuroradiology, University Hospital Augsburg, Augsburg, Germany; ^6^Department of Neurology, Bezirkskliniken Schwaben, Kaufbeuren, Germany

**Keywords:** stroke, hospitalizations, quality assurance, in-hospital mortality, pandemic

## Abstract

**Background and purpose:**

At the beginning of the SARS-CoV-2 pandemic, an alarming decline in hospitalizations for stroke was reported in several countries, including Germany. We assessed hospitalization numbers and indicators of the quality of stroke care in 2020 during the pandemic containment measures.

**Materials and methods:**

The analysis was based on data of two large stroke quality assurance registries in the north and the south of Germany (Qualitätssicherung Schlaganfall Nordwestdeutschland and Bayerische Arbeitsgemeinschaft für Qualitätssicherung in der stationären Versorgung). We included 395 hospitals with 467,931 documented cases in 2018–2020. The time interval between admission and thrombolysis, frequency of systemic thrombolysis and intra-arterial therapy (IAT), National Institutes of Health Stroke Scale (NIHSS) score on admission and in-hospital mortality were assessed. Changes in the second (Q2) and fourth (Q4) quarters of 2020 were compared to corresponding quarters in 2019 by chi-squared tests.

**Results:**

Hospitalization numbers decreased in the two stroke registries by 8% and 10% in Q2 of 2020 and by 5% and 15% in Q4 of 2020 compared to the same quarters in 2019, respectively. The decline was particularly seen in women and patients with transient ischemic attacks. In cases with cerebral infarction, no increase in NIHSS scores on admission was observed, and the proportion of patients with a time interval between admission and thrombolysis of ≤60 min was unchanged. No clear pattern was found in the frequency of systemic thrombolysis and IAT. In one of the registries, in-hospital mortality of patients with cerebral infarction increased in Q2 of 2020 compared to Q2 of 2019.

**Conclusion:**

Case numbers slightly decreased under pandemic conditions, while our quarterly analysis indicated that the quality of stroke care was largely unchanged throughout the year 2020.

## Introduction

In the months following the onset of the SARS-CoV-2 pandemic (COVID-19), a decrease in the number of patients with stroke admitted to hospitals was observed in several countries (1, 2), which raised concerns about delays in adequate diagnosis and treatment of patients with stroke. Evaluation of a U.S. national inpatient stroke registry indicates a decline of acute ischemic stroke presentations of 15.3% in the period from the first week of February to the fourth week of June in 2020 when compared to the same weeks in 2019 (3).

In administrative data of hospitals in Germany for reimbursement purposes, a strong decline in the hospitalization of cases with acute ischemic stroke (−18.5%), transient ischemic attack (TIA, −26.1%), and intracerebral hemorrhage (−18.3%) was observed between 16 March 2020 and 15 May 2020 compared to the corresponding time period in 2019 (4). An analysis of health claims data from a large insurance company in Germany found declining admission rates of −8.9% for stroke and −14.6% for TIA from January to May 2020 compared to the same months in the previous year (5). A study of another health insurance focused on 3 weeks at the beginning of the first lockdown in Germany (16 March 2020 to 5 April 2020) and found a 15% decrease in hospitalizations for cerebral infarction or intracerebral hemorrhage and a 35% decrease for TIA when compared to 25 March 2019 to 14 April 2019 (6).

Especially at the beginning of the COVID-19 pandemic, strict social containment measures and fear of in-hospital infection may have caused patients, particularly those with minor symptoms, to delay or avoid seeking inpatient treatment. Furthermore, pandemic measures might have caused delays during the hospital admission procedure because of screening for infectious symptoms and the application of personal protective equipment, potentially delaying time-sensitive treatment options. Previous studies were often limited to the first pandemic wave, and most studies only analyzed admission frequencies. The selected time periods and the corresponding declines in hospital admissions varied substantially between studies (4–6). Analyses beyond describing frequencies, e.g., the inclusion of measures of quality of care or early outcomes, during the pandemic are scarce. In addition, longer periods of time with more than one pandemic wave to evaluate potential catch-up effects in hospitalizations are necessary to comprehensively interpret the effects of the pandemic on stroke care.

We analyzed the number of stroke cases in two large stroke registries in the South and the Northwest of Germany and evaluated evidence-based indicators of the quality of stroke care under pandemic conditions throughout the year 2020, with a particular focus on the second (Q2) and fourth (Q4) quarters, that were characterized by COVID-19 containment measures in Germany.

## Materials and methods

Two independent quality assurance stroke registries, one in Northwestern Germany (Qualitätssicherung Schlaganfall Nordwestdeutschland, quality assurance stroke register of northwestern Germany, QSNWD) and one in the South of Germany (Bayerische Arbeitsgemeinschaft für Qualitätssicherung in der stationären Versorgung, Bavarian Association for Quality assurance of inpatient care, BAQ), were analyzed separately in this study. Both registries are members of the German Stroke Registers Study Group (Arbeitsgemeinschaft Deutschsprachiger Schlaganfall-Register, ADSR), a voluntary network of 10 regional quality assurance projects. The German Stroke Registers Study Group ADSR was founded in 1999 to document the quality of acute stroke care with standardized data collection methods and definitions of performance measures of stroke care (7, 8).

### Data collection and inclusion criteria

Hospitalizations of patients with stroke were documented in departments of neurology and internal medicine of academic and community hospitals in urban and rural regions. In this study, we assessed all documented cases of TIA (ICD-10 G45), cerebral infarction (ICD-10 I63), cerebral hemorrhage (ICD-10 I61), and stroke, not specified as hemorrhage or infarction (ICD-10 I64) that occurred between 2018 and 2020. Data, for e.g., sociodemographic information, symptoms, the time interval from event to admission, treatments, and complications, were collected with standardized data collection forms. The standardized data assessments facilitated the high comparability of the two registries.

For the QSNWD registry, 163 hospitals that continuously documented stroke cases between 2017 and 2020 were included. These hospitals are located in ten different German federal states. A list of the included hospitals is provided in Supplementary Table 1. Participation in the QSNWD registry is voluntary, though hospitals with a stroke unit have to prove quality assurance by a registry to be certified by the German Stroke Society. Data from the included cases were mainly collected electronically (93.7%), but data collection was also facilitated on paper (6.3%) (9). Forms on paper were scanned and checked for completeness and plausibility. Inadvertently submitted forms and duplicate cases due to multiple data uploads to the registry were removed. In the QSNWD registry, 321,505 hospitalizations during the study period 2018–2020 were documented for patients with TIA, cerebral infarction, cerebral hemorrhage, and stroke, not specified as hemorrhage or infarction.

For the BAQ registry, 232 hospitals located in Bavaria were considered (Supplementary Table 1). Documentation of hospitalized patients with stroke was mandatory in Bavaria. Data were collected completely electronically and checked for plausibility and completeness (10). We considered 146,426 documented hospitalizations in the BAQ registry in 2018–2020 of patients with the diagnoses specified above.

Anonymized datasets were used for this analysis, and data collection was done as part of the stroke quality assurance program in the participating hospitals. Data protection regulations restricted analysis of the number of hospital admissions and quality of stroke care to a quarterly time resolution. We especially considered the second (Q2; April 1 to June 30) and fourth (Q4; October 1 to December 31) quarters of 2020 because these quarters were particularly affected by COVID-19 containment measures, even though the first nationwide containment measures in Germany started already toward the end of the first quarter on 23 March 2020. Documentation of a stroke case in the register refers to one specific hospitalization of a patient. If the same patient is admitted to a hospital several times or transferred to another hospital, the different hospital admissions appear as separate cases with individual identification numbers in the registry.

### Quality indicators and variables

We evaluated three quality indicators according to the German Stroke Registers Study Group ADSR definition (11) (status on 4 March 2021), which we considered particularly important performance measures of stroke care because of their time dependency. These parameters provide useful information on whether the pandemic was associated with a delay in seeking inpatient treatment for stroke: (1) The proportion of patients treated with systemic thrombolysis was calculated in cases with cerebral infarction and a time interval event-to-admission of ≤4 h, a National Institutes of Health Stroke Scale (NIHSS) score of 4–25, and an age of 18–80 years. Patients with intra-arterial therapy (IAT), patients transferred for systemic thrombolysis to another hospital, patients with systemic thrombolysis before admission, and patients with in-house stroke were excluded. (2) IAT was assessed in patients with cerebral infarction and vessel occlusion (carotid-bifurcation, middle cerebral artery M1 segment, and basilar artery) and a time interval event-to-admission of ≤6 h or in-house stroke. Patients with IAT before admission and patients transferred to another hospital for IAT were excluded. (3) The proportion of cases with a time interval between admission and thrombolysis of ≤60 min was calculated in patients with cerebral infarction, intravenous thrombolysis, a time interval event-to-admission of ≤4 h (including in-house stroke), an NIHSS score of 4–25, and an age of 18–80 years. Cases transferred for thrombolysis were not considered for this quality indicator.

Further quarterly frequencies for all included cases were calculated for diagnosis, sex, age, the living situation before admission (independent at home, care at home, and nursing home), the duration of stay in the hospital, and in the stroke unit. Moreover, we assessed the NIHSS score on admission in patients with cerebral infarction categorized into minor stroke (NIHSS 0– 3) and major stroke (NIHSS 4–42) (12). For all included cases, we determined the Barthel index on admission classified into a higher need of care (Barthel index 0–75) and lower need of care (Barthel index >75) as well as the time interval from event to admission categorized as ≤1 h, >1–2 h, >2–3 h, >3–4 h, >4–5 h, >5–6 h, >6–24 h, >24–48 h, >48 h−7 days, in-house stroke, and unknown. In addition, we plotted quarterly frequencies of the complications of bleeding and pneumonia. Changes in the case mix within a quarter could influence the proportion of deceased patients, e.g., due to a reduced number of hospitalized cases with TIA. Therefore, we focused our examination of deaths in hospitals on cases with cerebral infarction.

### Statistical analysis

We calculated absolute and relative frequencies with confidence limits separately for both registries. The three considered time-dependent quality indicators were assessed in the subgroups defined above. Changes in percentages from Q2 of 2019 to Q2 of 2020 and from Q4 of 2019 to Q4 of 2020 were examined by chi-squared tests with α < 0.05. As our datasets comprise large numbers of cases, even small differences can appear statistically significant. Therefore, the clinical relevance of changes has to be contextualized. Missings were excluded from the respective analyses except the variable “time interval from event to admission,” which comprised the category “unknown.” In the QSNWD registry, the number of missing information for the other variables ranged from 0.05% in systemic thrombolysis to 8.06% in the time interval from event to admission. In the BAQ registry, most of the variables had no missing values; however, missing information ranged from 2.59% in the Barthel index on admission. We assessed possible pre-pandemic trends in 2018–2019 in the number of cases and in-hospital mortality using the Mann–Kendall test and the Cochran–Armitage trend test, respectively.

The datasets of QSNWD and BAQ were analyzed separately to show the replicability of results from two independent registries. No data linkage across databases was performed. The investigators, AB and VR, performed the analysis and had full access to the database of the respective population. Analyses were carried out using SAS 9.4 (SAS Institute Inc., Cary, NC).

## Results

In the 163 included hospitals in Northwestern Germany, 324,229 stroke cases were admitted between 2018 and 2020, of which we excluded 2,724 cases with subarachnoid hemorrhage (ICD-10 I60) or a diagnosis other than TIA, cerebral infarction, cerebral hemorrhage, or stroke, not specified as hemorrhage or infarction. Thus, 321,505 cases from the QSNWD registry were included. In the BAQ registry, covering 232 hospitals in the federal state of Bavaria, 147,260 cases were admitted in the period 2018–2020. As 834 cases with subarachnoid hemorrhage were excluded, 146,426 cases from the BAQ registry with a diagnosis of ICD-10 G45, I63, I61, or I64 were analyzed.

**Figure 1:**
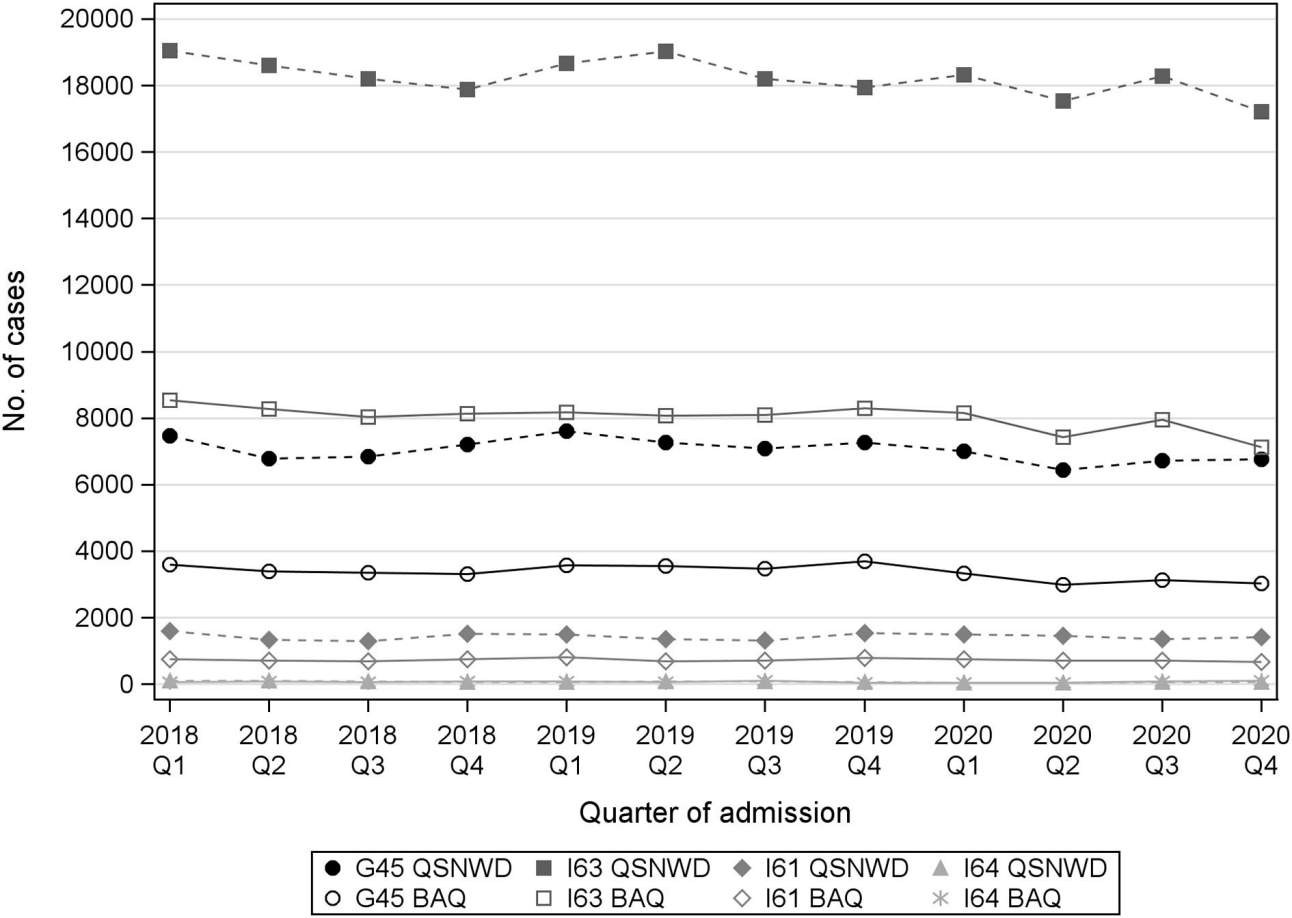
Number of cases with transient ischemic attack (ICD-10 G45), cerebral infarction (ICD-10 I63), cerebral hemorrhage (ICD-10 I61), or stroke, not specified as hemorrhage or infarction (I64) from the registries QSNWD (dashed lines) and BAQ (solid lines) in 2018–2020 by quarter of admission.

In the QSNWD registry, the number of documented cases was 8% lower in the second quarter of 2020 (*n* = 25,473) during the first COVID-19 lockdown compared to the respective quarter in the previous year (*n* = 27,736 in Q2 of 2019; Supplementary Table 2). In the fourth quarter of 2020, the number of cases was 5% lower (*n* = 25,461 in Q4 of 2020 vs. *n* = 26,807 in Q4 of 2019). The BAQ registry showed slightly stronger declines of 10% (*n* = 11,174 in Q2 of 2020 vs. *n* = 12,396 in Q2 of 2019) and 15% (*n* = 10,939 in Q4 of 2020 vs. *n* = 12,826 in Q4 of 2019; Supplementary Table 3). Case numbers decreased especially for cerebral infarction and TIA (Figure 1, Supplementary Tables 2, 3), while no pre-pandemic trend in 2018–2019 was identified. In the QSNWD registry, hospitalizations in Q2 and Q4 of 2020 were 7.8 and 3.9%, respectively, lower for cerebral infarction and 11.3% and 6.9% lower for TIA, respectively. A stronger decline was seen in the BAQ registry with 8.1% and 14.1% reduced hospitalizations for cerebral infarction and 16.0% and 17.8% for TIA in Q2 and Q4 of 2020, respectively. This decline was more pronounced in women (Supplementary Figures 1, 2).

The NIHSS scores on admission in patients with cerebral infarction were not elevated in Q2 and Q4 of 2020 (Supplementary Tables 2–4). In the QSNWD registry, the proportion of patients with cerebral infarction who have an NIHSS score on the admission of ≥4 only slightly, but statistically significantly, decreased (Q2: −1.82 p.p., *p* = 0.001; Q4: −1.91 p.p., *p* ≤ 0.001). Furthermore, not any notable changes were apparent for the Barthel index and the living situation before admission as well as for sex, age, and the time interval between event and admission. The proportion of patients with a Barthel index on the admission of ≤75 showed a statistically significant decrease of −1.18 p.p. (*p* = 0.01) in the QSNWD registry and of −1.81 p.p. (*p* = 0.01) in the BAQ registry in Q4 of 2020. In both registries, the proportion of women decreased marginally, but statistically significantly, in Q4 of 2020 (QSNWD: −1.07 p.p., *p* = 0.015; BAQ: −1.6 p.p., *p* = 0.014). The percentage of unknown event-to-admission times ranged from 7.2 to 8.5% in 2019–2020. The median duration of stay in the hospital was 1 day lower in Q4 of 2020 compared to Q4 of 2019 in both registries. For the QSNWD registry, the median duration of stay in the stroke unit was evaluated additionally and decreased from 3 days in Q4 of 2019 to 2 days in Q4 of 2020.

**Figure 2:**
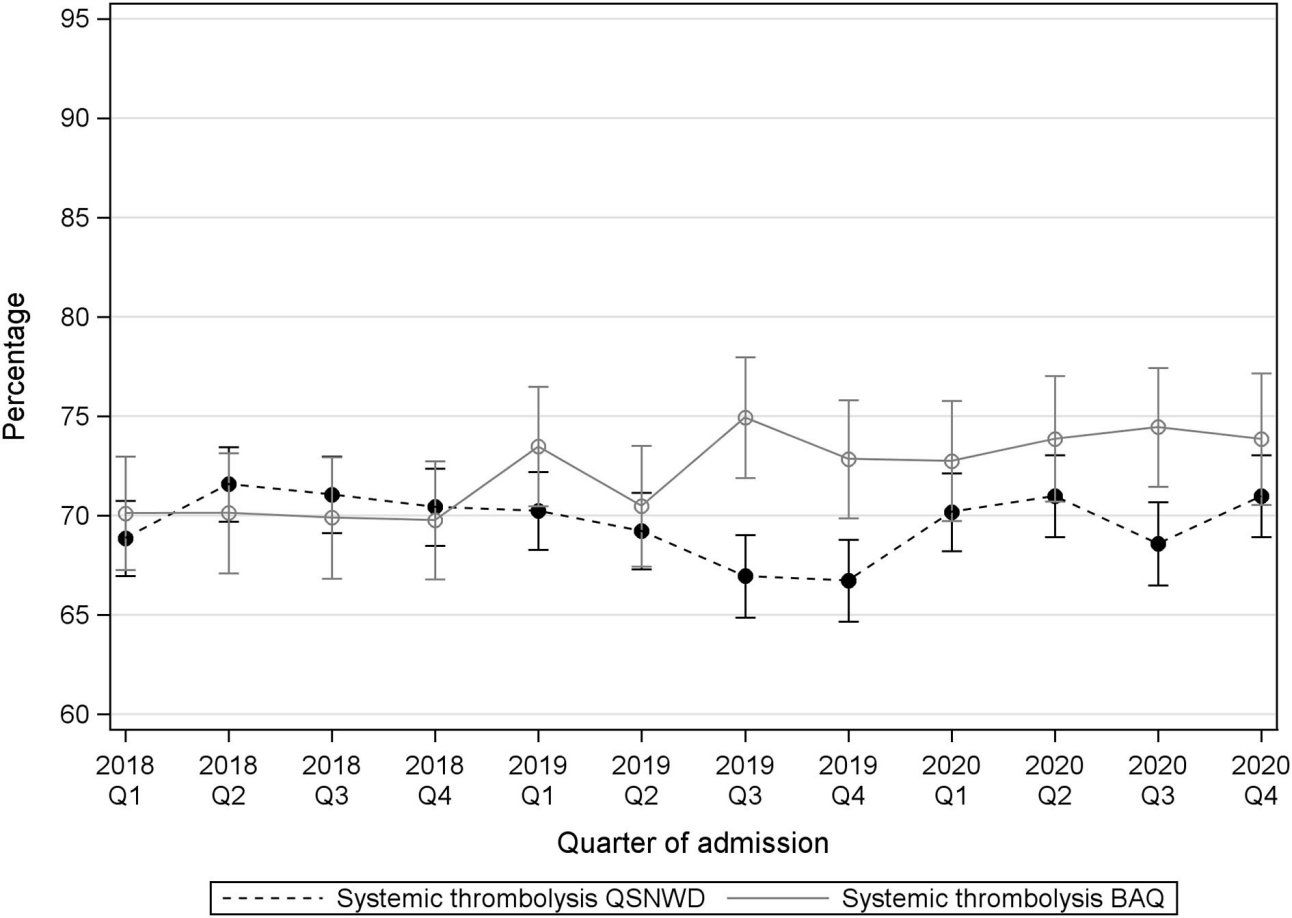
Systemic thrombolysis in patients with cerebral infarction, a time interval event to admission of ≤4 h, a NIHSS 4–25 and 18–80 years old from the registries QSNWD (dashed line) and BAQ (solid line) in 2018-2020 by quarter of admission.

**Figure 3:**
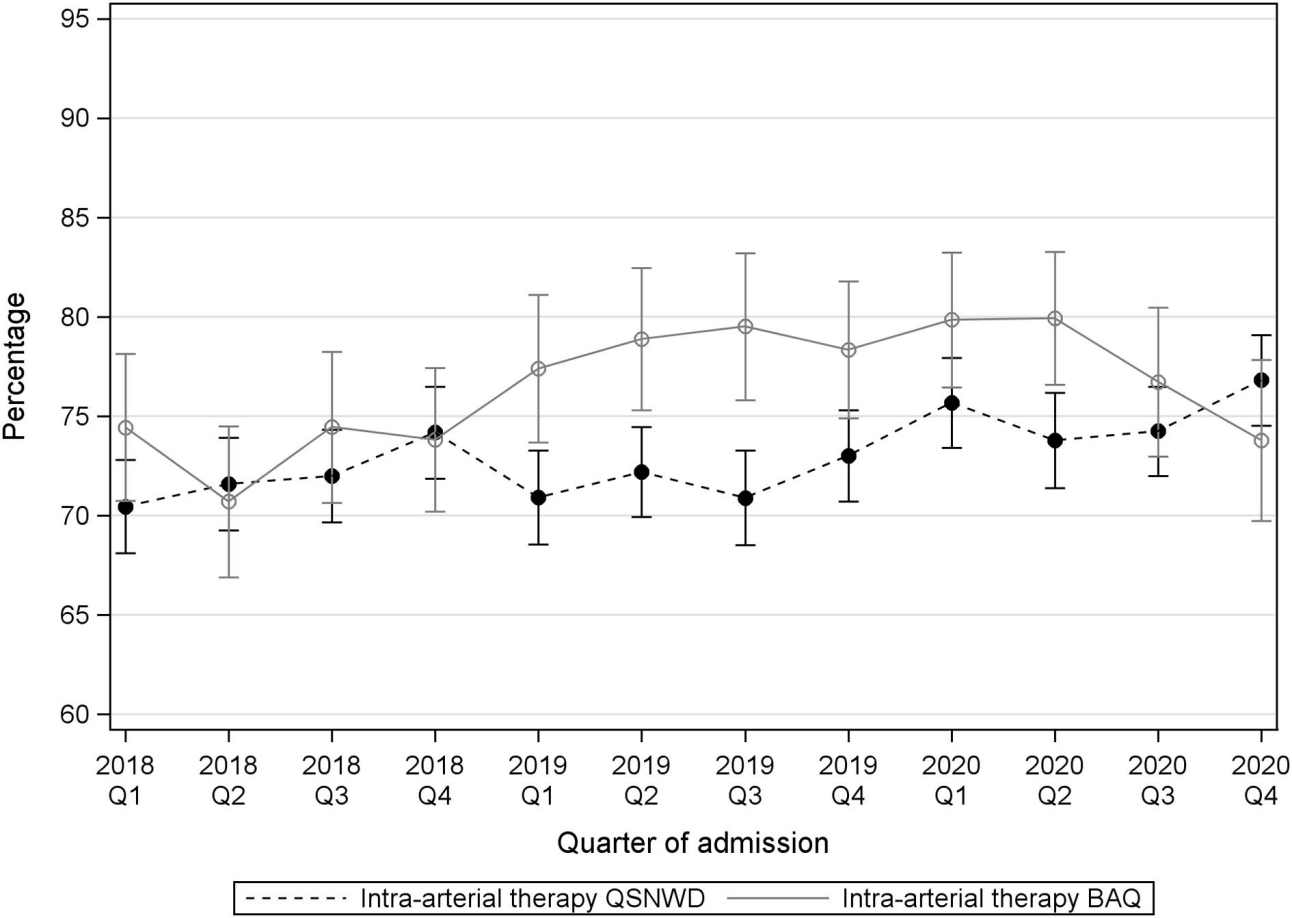
Intra-arterial therapy (IAT) in patients with cerebral infarction, vessel occlusion, and a time interval event to admission of ≤6 h or in-house stroke from the registries QSNWD (dashed line) and BAQ (solid line) in 2018-2020 by quarter of admission.

The proportion of cases with systemic thrombolysis varied slightly by quarter, but no clear pattern appeared over time (Figure 2). In the QSNWD registry, the frequency of systemic thrombolysis increased statistically significantly by 4.2% points (p.p.) from 66.7% in Q4 of 2019 to 71.0% in Q4 of 2020 (*p* = 0.004), whereas changes in the BAQ registry were not statistically significant (Supplementary Table 4). The frequency of IAT did not change considerably (Figure 3), but a statistically significant increase of 3.8 p.p. was seen in the QSNWD registry for the fourth quarter of 2020 (73.0% in Q4 of 2019 vs. 76.8 in Q4 of 2020, *p* = 0.02).

**Figure 4:**
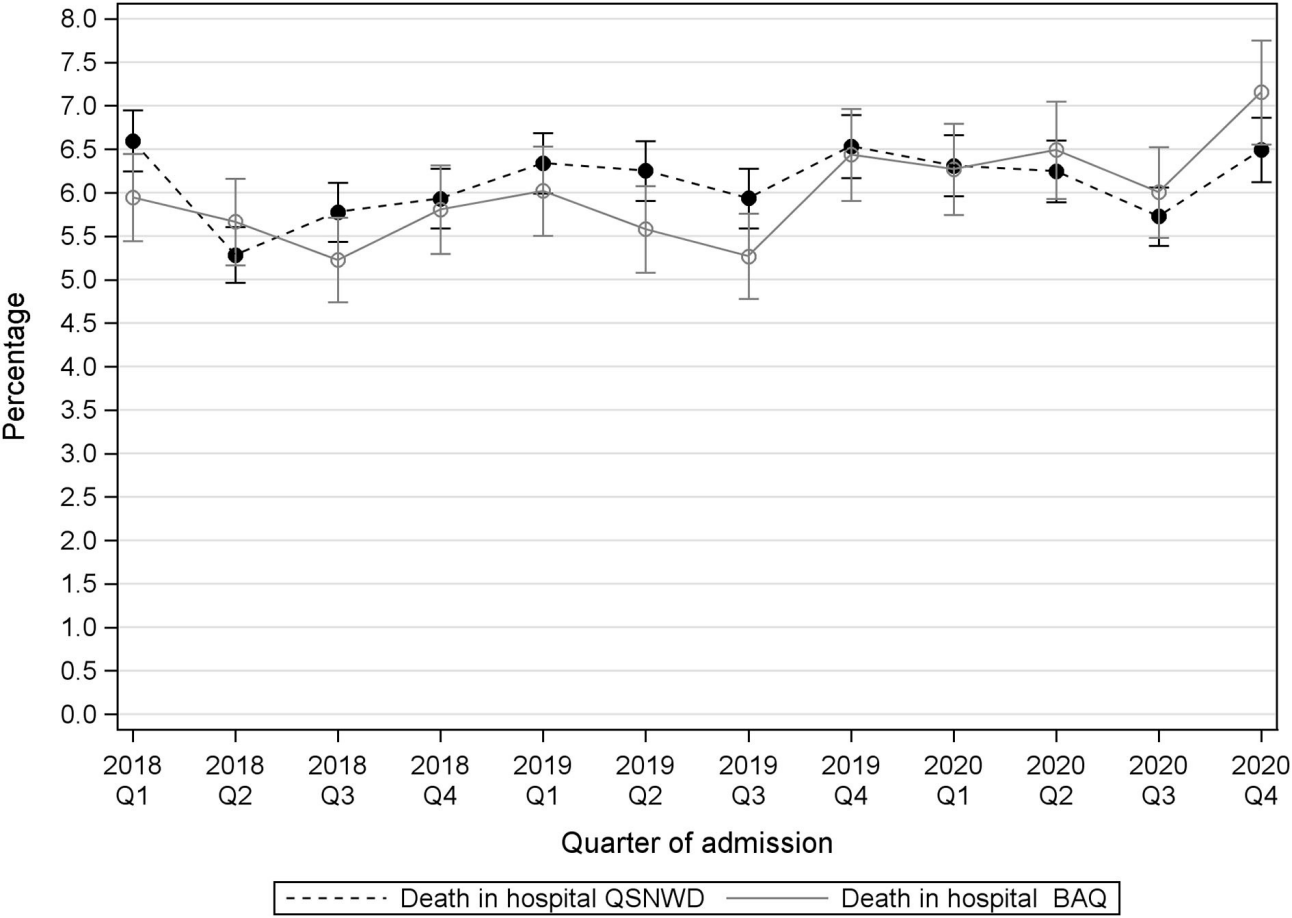
Death in hospitalized patients with cerebral infarction (ICD-10 I63) from the registries QSNWD (dashed line) and BAQ (solid line) in 2018–2020 by quarter of admission.

**Figure 5:**
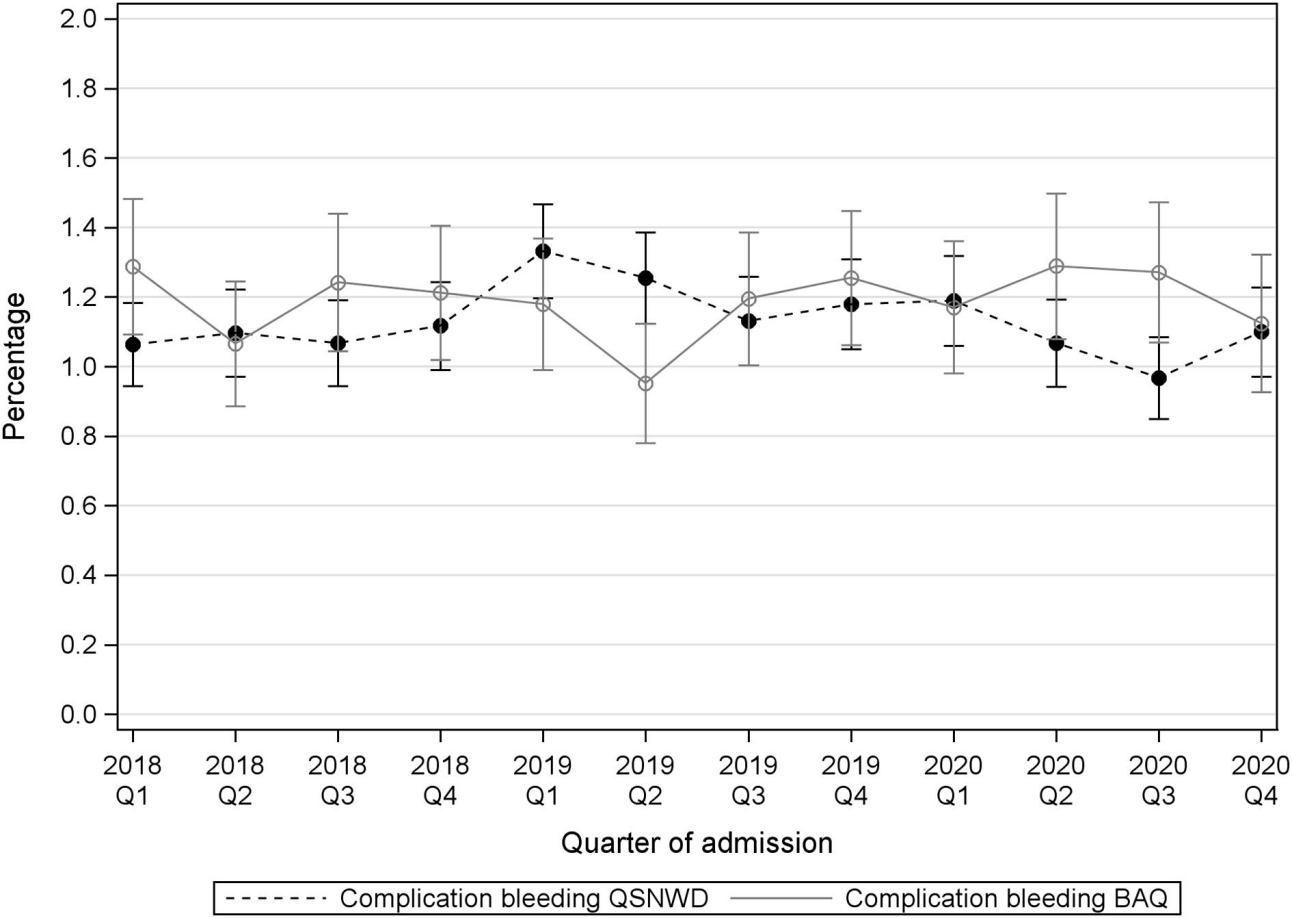
Complication bleeding of all included patients from the registries QSNWD (dashed line) and BAQ (solid line) in 2018-2020 by quarter of admission.

**Figure 6:**
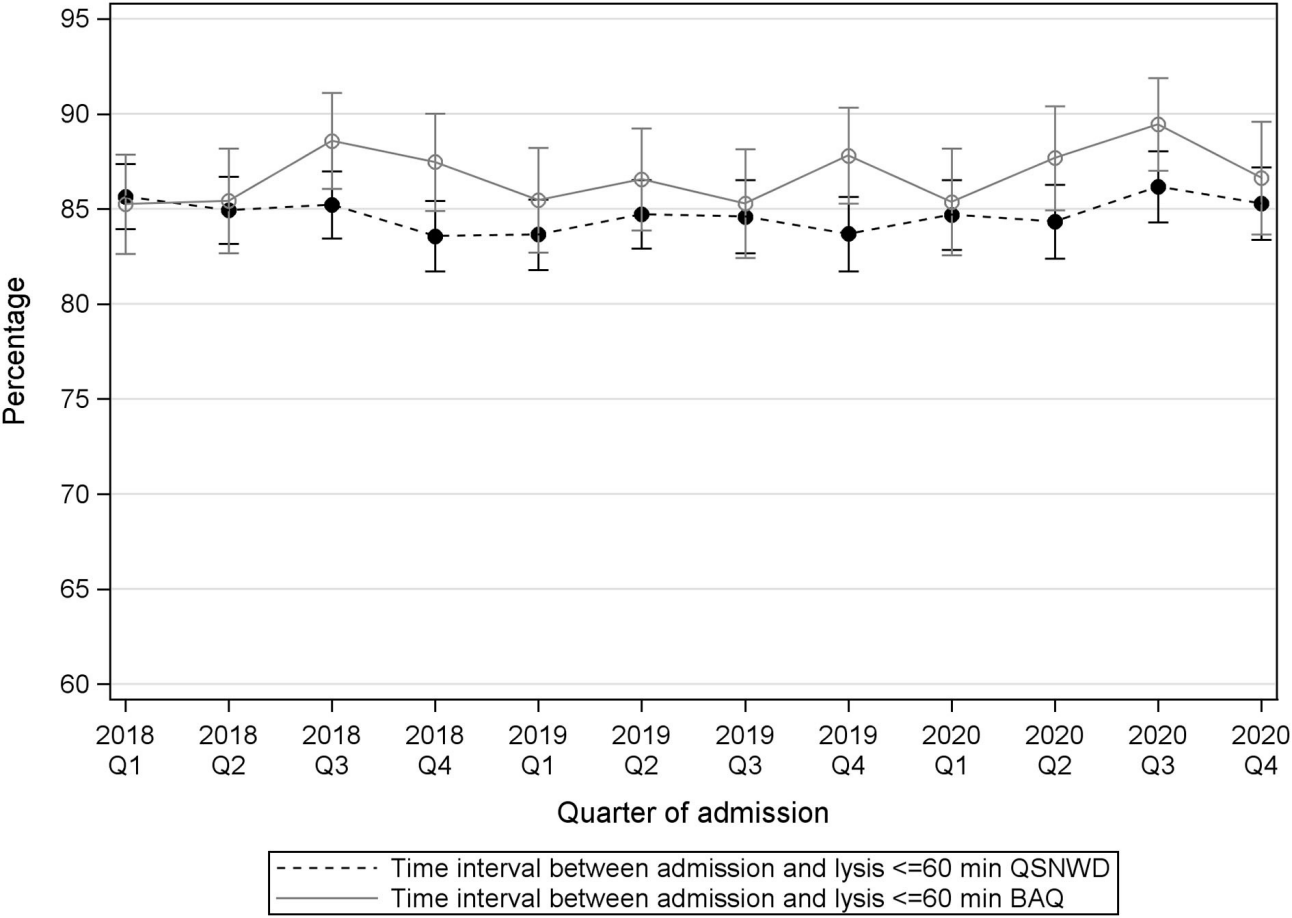
Time interval between admission and thrombolysis ≤60 min (in the same house) in patients with cerebral infarction, intravenous thrombolysis, a time interval event to admission of ≤4 h (including in-house stroke), a NIHSS 4–25 and 18–80 years old from the registries QSNWD (dashed line) and BAQ (solid line) in 2018-2020 by quarter of admission.

The percentage of patients with cerebral infarction that deceased in the hospital varied during the assessed 3 years (Figure 4). A statistically significant positive trend in the pre-pandemic period from 2018 to 2020 was only found in the QSNWD registry (*p* = 0.049), whereas during the pandemic, in-hospital mortality of patients with cerebral infarction was not elevated. In contrast, no statistically significant pre-pandemic trend was found in the BAQ registry (*p* = 0.4107), but a statistically significant increase of 0.9 p.p. from 5.6% in Q2 of 2019 to 6.5% in Q2 of 2020 (*p* = 0.017) was observed and in-hospital mortality was highest in Q4 of 2020 with 7.16%. Cerebral bleeding as a complication was fairly constant over time (Figure 5). However, small opposite changes were found in both registries (QSNWD: −0.18 p.p. from 1.25% in Q2 of 2019 to 1.07 in Q2 of 2020, *p* = 0.045; BAQ: +0.34 p.p. from 0.95% in Q2 of 2019 to 1.29% in Q2 of 2020, *p* = 0.014). The proportion of patients with a time interval between admission and lysis (also known as door-to-needle time) of ≤60 min was quite consistent (Figure 6). Likewise, the frequency of pneumonia was rather steady, but only a statistically significant increase of 0.54 p.p. from 3.65% in Q2 of 2019 to 4.19% in Q2 of 2020 (*p* = 0.035) was observed in the BAQ registry (Supplementary Figure 3).

## Discussion

In the stroke registries, QSNWD and BAQ, the overall number of documented cases decreased by 8% and 10% in Q2 of 2020 and by 5% and 15% in Q4 of 2020, respectively, compared to the corresponding quarters of the preceding year. The decline was higher in the Bavarian registry (BAQ) and more pronounced among women. Contrary to our expectations, NIHSS scores upon admission in cases with cerebral infarction were not higher than in the comparison periods, and the proportion of patients with a time interval between admission and thrombolysis of ≤60 min was constant. The frequency of interventions (systemic thrombolysis or IAT) was also not different in 2020.

Preceding studies from German health claims and administrative hospital data indicated a sharp decrease in hospitalized stroke cases at the beginning of the pandemic (4, 6). However, the periods considered in those studies were short, selectively focused on the first pandemic wave, and within that wave, further restricted to the first 3 weeks (6) or 9 weeks (4) period during the complete lockdown in Germany. By contrast, we examined the whole year of 2020 and assessed quarterly changes. Therefore, shifts in the longer term including possible “catch-up effects” of hospitalizations are comprised. This is important because it was discussed in the literature that, if the reductions in cases were primarily represented by an absence of minor strokes and transient ischemic attacks, an influx of patients with stroke in the subsequent months could be expected due to a higher rate of secondary strokes in consequence of the lack of adequate secondary prophylaxis (13). In our study, this part could have balanced out the differences in hospitalizations and disease severity within the examined quarters. If such a “catch-up effect” was present within a certain quarter, a short-term change in that quarter would be discernable less clearly in a quarterly analysis compared to an analysis with a finer, weekly, or daily resolution of time periods.

Various reasons for the drop in hospital admissions are conceivable. Social and physical distancing measures and anxiety of getting infected with the coronavirus could have especially affected patients with TIA, because consultation with family members and general practitioners before a hospital admission might play a role, especially in patients with mild symptoms. The stronger decline of hospitalization in women could partly be explained by the fact that older women are a vulnerable population because they commonly remain living alone as “surviving spouses.” Increased social isolation could have hindered family members from noticing milder, transitory symptoms of the elderly. On the contrary, family members could also have spent more time together at home during the lockdowns and, therefore, might recognize stroke symptoms earlier. Hence, opposite effects could be possible. The more pronounced decline of hospitalization in women is in line with the results of a recent analysis of German nationwide administrative hospital data. Richter et al. found a greater decline in hospital admissions of female patients compared to male patients with acute ischemic stroke during both the first and the second pandemic waves (14).

Although we observed a slight reduction of documented cases with cerebral infarction, their stroke severity measured by the NIHSS upon admission did not increase. According to this finding, a similar proportion of cases with minor symptoms were hospitalized during the COVID-19 lockdowns. This is in accordance with the analysis of U.S. national inpatient stroke registry data on 81,084 patients with acute ischemic stroke who showed decreased hospitalizations and consistent median NIHSS scores during COVID-19 (4 February 2020 to 29 June 2020) compared to pre-pandemic period (1 November 2019 to 3 February 2020) (3). In contrast, an analysis of German health claims data found that patients with cerebral infarction or intracerebral hemorrhage more frequently suffered from hemiparesis or hemiplegia, speech impairment, swallowing difficulties, and hemispatial neglect (6). This was seen in a 3-week period at the beginning of the first lockdown compared to a corresponding period in 2019.

Our finding of a rather consistent proportion of patients with a time interval between admission and thrombolysis of ≤60 min is in line with a recent systematic review and meta-analysis on the impact of the pandemic on stroke care across the world, which identified 46 studies including 129,491 patients. The authors found no differences in the mean door-to-needle time between the COVID-19 pandemic and the pre-pandemic era (1). Likewise, in the U.S. national inpatient stroke registry, which is voluntarily used by more than 2,000 hospitals in the United States, patients with acute ischemic stroke had similar door-to-needle times in the COVID-19 time period compared to the pre-COVID-19 era (3). Assessment of this quality indicator portends that precautionary measures, such as tests for COVID-19 and carrying of additional personal protective equipment, seem not to have delayed patient care.

The quality indicators “frequency of systemic thrombolysis and IAT” also showed no change in 2020 compared to the years before. If at all, a small increase was seen in the QSNWD registry in Q4 of 2020. The above-mentioned meta-analysis of Katsanos et al. (1) found no difference in the rates of intravenous thrombolysis administration, but a higher probability of receiving endovascular thrombectomy treatment during the COVID-19 pandemic. In administrative hospital data, Richter et al. (4) also observed unchanged intravenous thrombolysis rates in patients with acute ischemic stroke, but a significantly higher mechanical thrombectomy rate during the pandemic. This was in agreement with an evaluation of German health claims data of large insurance (6), whereas Seiffert et al. (5) did not identify changed treatment allocations in claims data of another health insurance. Comparing IAT rates during the pandemic to periods in preceding years, it has to be considered that the frequency of mechanical thrombectomy treatment substantially increased within the last decade (15). The reason for this increase was new evidence published in 2015, which resulted in modification of medical guidelines and changed indications for mechanical thrombectomy (16). The absence of an increase in the frequency of IAT in the BAQ registry in 2020 could be associated with higher COVID-19 case numbers in the South of Germany. Temporarily overloads of intensive care units might have required transfers of patients with stroke potentially causing delays in treatment. However, we observed no increased time interval from event to admission in Q2 and Q4 of 2020 for the BAQ registry.

In-hospital mortality of patients with cerebral infarction varied over time in the QSNWD registry without any clear pattern associated with the COVID-19 lockdowns. However, in the BAQ registry, a statistically significant increase in in-hospital mortality by 0.9 p.p. was observed in Q2 of 2020 compared to Q2 of 2019, and it was highest in Q4 of 2020. This should be seen in the light of the variation during the assessed 3 years. The increase could be a temporary phenomenon, and changes become statistically significant more easily in large datasets. Furthermore, as deaths in hospital is a rather rare event and the BAQ registry is less than half the size of the QSNWD registry, random variation might be larger. The percentage of patients with cerebral infarction that deceased in the hospital was not adjusted for case mix, age, sex, or NIHSS at admission and therefore the proportion of inevitable deaths is not clear. However, Barthel index, the living situation before admission, sex, age, and the time interval between event-to-admission did not change notably, and no increase of NIHSS at admission was found for cases with cerebral infarction, which indicates a rather consistent case mix. In the U.S. registry-based study, in-hospital mortality of patients with acute ischemic stroke was assessed, and no significant difference between a pre-pandemic period (1 November 2019 to 3 February 2020) and a pandemic period (1 April 2020 to 29 June 2020) remained when adjusted for patient demographics, clinical characteristics, medical history, and hospital characteristics (OR = 1.11, *p* = 0.03) (3). The meta-analysis of Katsanos et al. (1) found an overall statistically significantly higher risk of in-hospital mortality (OR = 1.26, *p* < 0.001) for patients with stroke during the pandemic periods compared to patients in the pre-pandemic periods, as defined in each included study. Additionally, Seiffert et al. (5) showed in their study on German health claims data that in-hospital mortality of patients with stroke statistically significantly increased from 8.5% in a pre-pandemic period (January to May 2019) to 9.8% in a pandemic period (January to May 2020) by 1.3 p.p. (*p* < 0.05). Evaluation of claims data on patients with cerebral infarction or intracerebral hemorrhage from another large health insurance demonstrated an increase in in-hospital mortality from 8.6% in a pre-pandemic period (25 March 2019 to 14 April 2019) to 10.9% in a pandemic period (16 March 2020 to 5 April 2020) by 2.3 p.p. (*p* < 0.001), and the 30-day mortality increased from 11.97% to 14.86% by 2.89 p.p. (*p* < 0.001) in the compared periods (6). Richter et al. (4) found in their analysis of administrative data of hospitals in Germany an increase in in-hospital mortality for patients with acute ischemic stroke from 7.4% in a pre-pandemic period (16 March 2019 to 15 May 2019) to 8.1% during the pandemic (16 March 2020 to 15 May 2020) by 0.7 p.p (*p* < 0.001). Changes in in-hospital mortality vary between studies and may depend on the length of the pandemic period assessed. Although assessment of 30-day mortality would be more comprehensive, our analysis was confined to in-hospital mortality because the documentation for the quality assurance registries ends with discharge.

The main limitation of this study is the coarse resolution of time in quarters. Due to data protection regulations, the exact date of hospital admission is not included in the anonymized datasets of the stroke registries. As the beginning of the first pandemic wave in Germany overlaps the change from the first to the second quarter of the year 2020, a short-term decline in hospitalizations might be less apparent in our quarterly analysis compared to a weekly or daily examination. Another limitation refers to the completeness of hospitalized stroke cases as documentation was not legally obliged in one of the participating registries. However, we only included hospitals that submitted data without interruption to this registry between 2017 and 2020 to ensure a certain continuity of documentation. Also, during the pandemic, hospital staff responsible for the documentation could have been overloaded causing a lack or delay in data entry. The latter would affect case numbers, especially in Q4 of 2020 toward the end of the observation period. Thus, a discrepancy between the number of documented cases submitted to the registry and the actual number of hospitalized cases cannot be ruled out in the QSNWD registry. Furthermore, we observed some very small changes, e.g., in sex distribution or frequency of IAT without clinical relevance but the changes appeared statistically significant because of the size of our datasets.

Despite these limitations, our study has several strengths. Our study provides a more comprehensive insight for a longer period than an analysis of selected weeks during the pandemic including potential catch-up effects after the end of the first wave. As case numbers possibly vary from year to year, we assessed the period 2018–2020 to put the changes in Q2 and Q4 of 2020 in the larger context of the two preceding years. In contrast, studies with a selective comparison of a short pandemic period with either a preceding pre-pandemic period or a corresponding short period in 2019 could partly be affected by temporal variations that are independent of the pandemic. An advantage of our analysis compared to previous studies on administrative data of hospitals for reimbursement purposes or health claims data from insurance companies is the more detailed information on the individual cases, e.g., regarding disease severity by the NIHSS, comorbidities, and the living situation before admission. Furthermore, we assessed quality indicators that are well-established and reported on a regular basis within the quality assurance of stroke care. Treatments, outcomes, and quality indicators were examined for the entire year 2020 covering both the first and the second pandemic waves. Moreover, our datasets comprise the NIHSS score to describe disease severity. Data collection and definition of quality indicators were standardized and unmodified during the observation period which allowed for replication of results from the two independent stroke registries. Another strength is given by the large database with an overall 467,931 included cases in 2018–2020 from academic and community hospitals in urban and rural regions.

## Conclusion

The number of hospitalized cases in two large German stroke registries decreased only slightly under pandemic conditions in Q2 and Q4 of 2020 compared to the respective quarters in 2019. Our quarterly analysis of quality indicators suggests that stroke care has been well preserved throughout the year 2020, and the time between admission and thrombolysis was not prolonged despite precautionary measures against COVID-19. However, although patients with cerebral infarction did not present with more severe symptoms, in-hospital mortality slightly increased in one of the registries. Future studies should continue monitoring stroke care and in-hospital mortality of patients with cerebral infarction during the further course of the COVID-19 pandemic in Germany. Case numbers might increase prospectively as patients who avoided hospital admission and did not receive adequate secondary prophylaxis subsequently could have an increased risk of a major stroke.

## Data availability statement

The data analyzed in this study is subject to the following licenses/restrictions: The data can be made available to any researcher for the express purpose of reproducing the here presented results, based on a written data transfer agreement and with the explicit permission for data sharing by the local institutional review board. Requests to access these datasets should be directed to Klaus Berger, bergerk@uni-muenster.de and Mario Callies, callies@baq-bayern.de.

## Ethics statement

Ethical review and approval was not required for the study on human participants in accordance with the local legislation and institutional requirements. Written informed consent for participation was not required for this study in accordance with the national legislation and the institutional requirements.

## Author contributions

AB and VR made the statistical analysis and wrote the first draft. AB, VR, PH, and KB contributed to the study conception and design. All authors commented on previous versions of the manuscript and approved the final manuscript.

## Conflict of interest

The authors declare that the research was conducted in the absence of any commercial or financial relationships that could be construed as a potential conflict of interest.

## Publisher's note

All claims expressed in this article are solely those of the authors and do not necessarily represent those of their affiliated organizations, or those of the publisher, the editors and the reviewers. Any product that may be evaluated in this article, or claim that may be made by its manufacturer, is not guaranteed or endorsed by the publisher.

## Abbreviations

BAQ: Bavarian Association for Quality assurance of inpatient care [Bayerische Arbeitsgemeinschaft für Qualitätssicherung in der stationären Versorgung]
COVID-19: SARS-CoV-2 coronavirus disease 2019
IAT: intra-arterial therapy
ICD-10: International Classification of Diseases (ICD) (10th version)
TIA: Transient ischemic attack
NIHSS: National Institutes of Health Stroke Scale
p. p.: Percentage points
QSNWD: quality assurance stroke register of northwestern Germany [Qualitätssicherung Schlaganfall Nordwestdeutschland].
