# Supplementary material for: Stroke care during the COVID-19 pandemic: Case numbers, treatments, and mortality in two large German stroke registries
Source: Front Neurol. 2022 Jul 22;13:924271. doi: 10.3389/fneur.2022.924271 (PMC9367687; doi:10.3389/fneur.2022.924271)
Supplement: Supplementary file 1 [file Data_Sheet_1.PDF]

Supplementary Material

Supplement Table 1 Name and place of included hospitals that contributed to the quality assurance stroke registries of Northwestern Germany (QSNWD) and of the Bavarian Working Party for Quality Assurance (BAQ)

**QSNWD registry**	
AMEOS Diakonie-Klinikum, Ueckermünde	Heinrich-Braun-Krankenhaus Zwickau, Zwickau
AMEOS Klinikum Bernburg, Bernburg	Helios Kliniken Aue, Aue
Agaplesion Diakonieklinikum Rotenburg gGmbH, Rotenburg	Helios Kliniken Schwerin, Schwerin
Agnes-Karll-Krankenhaus Laatzen, Laatzen	Helios Klinikum Bad Saarow, Bad Saarow
Allgemeines Krankenhaus Celle, Celle	Helios Klinikum Erfurt, Erfurt
Ammerland Klinik GmbH, Westerstede	Helios Klinikum Hildesheim GmbH, Hildesheim
Asklepios Fachklinikum Brandenburg, Brandenburg	Helios Klinikum Wuppertal-Barmen, Wuppertal
Asklepios Fachklinikum Lübben, Lübben	Helios-Klinikum Gotha, Gotha
Asklepios Fachklinikum Teupitz, Teupitz	Herz-Jesu-Krankenhaus, Münster-Hiltrup
Asklepios Klinik Pasewalk, Pasewalk	Hufeland Klinikum GmbH Standort Mühlhausen, Mühlhausen
Asklepios Klinikum Uckermark, Schwedt	Immanuel Klinik Rüdersdorf, Rüdersdorf
Asklepios-Kliniken Schildautal, Seesen	Johannes Wesling Klinikum Minden, Minden
BG Universitätsklinikum Bergmannsheil GmbH, Bochum	KKRN Katholisches Klinikum Ruhrgebiet Nord GmbH, Marl
BG-Kliniken Bergmannstrost, Halle (Saale)	KMG Klinikum Güstrow GmbH, Güstrow
CaritasKlinikum Saarbrücken, Saarbrücken	KRH Klinikum Nordstadt, Hannover
Carl-Thiem-Klinikum, Cottbus	Katholisches Krankenhaus St. Johann Nepomuk, Erfurt
Centre Hospitalier de Luxembourg, LUXEMBOURG	Kliniken Erlabrunn gGmbH, Breitenbrunn
Christliches Klinikum Melle, Melle	Kliniken Maria Hilf, Mönchengladbach
Christliches Krankenhaus, Quakenbrück	Klinikum Altenburger Land GmbH, Altenburg
Christophorus-Kliniken GmbH, Dülmen	Klinikum Arnsberg GmbH -St. Johannes Hospital-, Arnsberg
DIAKOVERE Friederikenstift, Hannover	Klinikum Bad Salzungen GmbH, Bad Salzungen
DIAKOVERE Henriettenstift, Hannover	Klinikum Barnim GmbH, Werner Forßmann KH, Eberswalde
DRK Krankenhaus Saarlouis, Saarlouis	Klinikum Braunschweig, Braunschweig
Diakonie Klinikum Neunkirchen gGmbH, Neunkirchen/Saar	Klinikum Bremen-Mitte, Bremen
Diakoniekrankenhaus Chemnitzer LandDIAKOMED gGmbH, Hartmannsdorf	Klinikum Bremerhaven-Reinkenheide, Bremerhaven
Dietrich-Bonhoeffer-Klinikum, Neubrandenburg	Klinikum Burgenlandkreis GmbH, Naumburg
Eichsfeld Klinikum GmbH, Kleinbartloff OT Reifenstein	Klinikum Chemnitz gGmbH, Chemnitz
Elbe-Klinikum Stade, Stade	Klinikum Dorothea Christiane Erxleben GmbH, Wernigerode
Elblandkliniken Meißen, Meißen	Klinikum Dortmund gGmbH / Städtische Kliniken, Dortmund
Elisabeth-Krankenhaus, Recklinghausen	Klinikum Frankfurt (Oder) GmbH, Frankfurt (Oder)
Euregio-Klinik, Nordhorn	Klinikum Herford, Herford
Ev. Bathildiskrankenhaus Bad Pyrmont gGmbH, Bad Pyrmont	Klinikum Ibbenbüren, Ibbenbüren
Ev. Krankenhaus Bielefeld Johannesstift, Bielefeld	Klinikum Lippe-Lemgo, Lemgo
Ev. Krankenhaus Bielefeld gGmbH - Gilead I, Bielefeld	Klinikum Lüdenscheid, Lüdenscheid
Evangelisches Krankenhaus GmbH, Gelsenkirchen	Klinikum Magdeburg gGmbH, Magdeburg
Evangelisches Krankenhaus Oldenburg, Oldenburg	Klinikum Meiningen GmbH, Meiningen
Evangelisches Krankenhaus Unna, Unna	Klinikum Osnabrück, Osnabrück
Evangelisches Krankenhaus, Castrop-Rauxel	Klinikum Saarbrücken gGmbH, Saarbrücken
Evangelisches Krankenhaus, Hattingen	Klinikum St. Georg gGmbH, Leipzig
Evangelisches Krankenhaus, Herne	Klinikum Uelzen, Uelzen
Fachkrankenhaus Hubertusburg gGmbH, Wermsdorf	Klinikum Westfalen GmbH, Betriebsteil Knappschaftskrankenhaus, Dortmund
Gemeinschaftskrankenhaus Herdecke, Herdecke	Klinikum Wolfsburg, Wolfsburg
Gesundheit Nord - Klinikum Bremen Nord, Bremen	Knappschaftsklinikum Saar GmbH Krankenhaus Sulzbach, Sulzbach
Gesundheitszentrum Bitterfeld/Wolfen- Akad. Lehrkrankenhaus der Martin-Luther-Universität Halle-Wittenberg, Bitterfeld-Wolfen	Knappschaftskrankenhaus Püttlingen, Püttlingen
HELIOS Klinikum Gifthorn, Gifthorn	Knappschaftskrankenhaus, Bottrop
HELIOS Vogtland-Klinikum Plauen, Plauen	Knappschaftskrankenhaus, Recklinghausen
Hans-Susemihl-Krankenhaus, Emden	Krankenhaus Plau am See, Plau am See
Hanse-Klinikum Stralsund, Stralsund	Krankenhaus St. Elisabeth-Stift, Damme
Hanse-Klinikum Wismar GmbH, Wismar	Kreisklinikum Siegen, Siegen
Heidekreis-Klinikum GmbH, Soltau	Kreiskrankenhaus Freiberg gGmbH, Freiberg

Supplement Table 1 Name and place of included hospitals that contributed to the quality assurance stroke registries of Northwestern Germany (QSNWD) and of the Bavarian Working Party for Quality Assurance (BAQ) *(continued)*

**QSNWD registry**	
Kreiskrankenhaus Greiz GmbH, Greiz	St. Franziskus-Hospital, Ahlen
Kreiskrankenhaus Gummersbach, Gummersbach	St. Georg Klinikum Eisenach, Eisenach
Kreiskrankenhaus Prenzlau, Prenzlau	St. Johannes Hospital, Hagen
Kreiskrankenhaus Prignitz gemeinnützige GmbH, Perleberg	St. Josef-Hospital, Bochum
Kreiskrankenhaus Rudolf Virchow, Glauchau	St. Marien-Hospital GmbH, Lünen
Kreiskrankhaus Schleiz, Schleiz	St. Marien-Hospital, Borken
Ludmillenstift, Meppen	St. Marien-Hospital, Hamm
Marienhaus Klinikum Saarlouis-Dillingen, Dillingen	St. Vincenz Krankenhaus Paderborn, Paderborn
Marienkrankenhaus St. Wendel, St. Wendel	St. Vincenz Krankenhaus, Menden
Martha-Maria Krankenhaus Halle-Dölau gGmbH, Halle (Saale)	Städtisches Klinikum Dresden - Standort Friedrichstadt, Dresden
Martin Gropius Krankenhaus GmbH, Eberswalde	Städtisches Klinikum Görlitz, Görlitz
Martin-Luther-Universität Halle-Wittenberg, Halle (Saale)	Städtisches Klinikum Wolfenbüttel gGmbH, Wolfenbüttel
Medizinische Hochschule, Hannover	Städtisches Klinikum, Lüneburg
Mittelweser Kliniken GmbH, Nienburg	Sächsisches Krankenhaus Altscherbitz, Schkeuditz
Muldentalkliniken GmbHKrankenhaus Wurzen, Wurzen	Sächsisches Krankenhaus Arnsdorf, Arnsdorf
Nordwest-Krankenhaus Sanderbusch, Sande	Sächsisches Krankenhaus, Rodewisch
Oberhavel Kliniken GmbH - Klinik Hennigsdorf, Hennigsdorf	Südharz-Krankenhaus Nordhausen GmbH, Nordhausen
Paracelsus-Klinik Zwickau, Zwickau	Thüringen-Kliniken "Georgius Agricola", Rudolstadt
Rhein-Maas Klinikum GmbH - Betriebsteil Marienhöhe, Würselen	Universität Leipzig, Leipzig
Ruppiner Kliniken GmbH, Neuruppin	Universität Münster, Münster
SHG Klinikum Merzig, Merzig	Universität Rostock, Rostock
SRH Waldklinikum Gera, Gera	Universitätsklinikum Aachen, Aachen
SRH Zentralklinikum, Suhl	Universitätsklinikum Carl Gustav Carus Dresden, Dresden
Sana Kliniken, Duisburg	Universitätsklinikum Göttingen, Göttingen
Sana Kliniken, Lübeck	Universitätsklinikum Jena, Jena
Sana Klinikum Borna, Borna	Universitätsklinikum Knappschaftskrankenhaus Bochum, Bochum-Langendreer
Sana Krankenhaus Templin, Templin	Universitätsklinikum des Saarlandes, Homburg / Saar
Sofien- und Hufeland-Klinikum GmbH, Weimar	Universitätsmedizin Greifswald, Greifswald
St. Ansgar-Krankenhaus, Höxter	Waldkliniken Eisenberg / Waldkrankenhaus "Rudolf Elle" GmbH, Eisenberg
St. Barbara Hospital, Gladbeck	Zentralklinik Bad Berka GmbH, Bad Berka
St. Bernward Krankenhaus, Hildesheim	Ökumenisches Hainich Klinikum GmbH, Mühlhausen
St. Elisabeth-Hospital, Gütersloh	
**BAQ registry**	
310KLINIK GmbH, Nürnberg	Capio Franz von Prümmer Klinik, Bad Brückenau
Anregiomed Klinik Dinkelsbühl, Dinkelsbühl	Caritas-Krankenhaus St. Josef Regensburg, Regensburg
Anregiomed Klinik Rothenburg, Rothenburg	Chirurgisches Klinikum München Süd, München
Anregiomed Klinikum Ansbach, Ansbach	Clinic Neuendettelsau, Neuendettelsau
Arberlandklinik Viechtach, Viechtach	Deutsches Herzzentrum München, München
Arberlandklinik Zwiesel, Zwiesel	diako - Die Stadtklinik Augsburg, Augsburg
Asklepios Klinik Burglengenfeld, Burglengenfeld	Diakoniewerk München-Maxvorstadt, München
Asklepios Klinik Lindau, Lindau	Donau Isar Klinikum Deggendorf, Deggendorf
Asklepios Klinik Oberviechtach, Oberviechtach	Donau Isar Klinikum Dingolfing, Dingolfing
Asklepios Stadtklinik Bad Tölz, Bad Tölz	Donau Isar Klinikum Landau, Landau
Barmherzige Brüder Regensburg Paul Gerhardt Haus, Regensburg	Donau Ries Klinik – Donauwörth, Donauwörth
Benedictus Krankenhaus Feldafing, Feldafing	Donau Ries Klinik – Oettingen, Oettingen
Benedictus Krankenhaus Tutzing, Tutzing	Donau Ries Klinik - Stiftungskrankenhaus Nördlingen, Nördlingen
Berufsgenossenschaftliche Unfallklinik Murnau, Murnau	Donauklinik Neu-Ulm, Neu-Ulm
Bezirksklinikum Obermain Kutzenberg, Ebensfeld	Fachklinik Bad Heilbrunn, Bad Heilbrunn
Bezirksklinikum Obermain Kutzenberg Tagesklinik, Coburg	Fachklinik Enzensberg, Enzensberg
Bezirksklinikum Regensburg, Regensburg	Fachklinik Herzogenaurach, Herzogenaurach
Bezirkskrankenhaus Günzburg, Günzburg	Fachklinik Ichenhausen, Ichenhausen
Bezirkskrankenhaus Kaufbeuren, Kaufbeuren	Gemeinnützige Krankenhausgesellschaft des Landkreis Bamberg Juraklinik Scheßlitz, Scheßlitz
Bezirkskrankenhaus Mainkofen-Passau Bezirksklinikum, Mainkofen	

Supplement Table 1 Name and place of included hospitals that contributed to the quality assurance stroke registries of Northwestern Germany (QSNWD) and of the Bavarian Working Party for Quality Assurance (BAQ) *(continued)*

**BAQ registry**	
Gemeinnützige Krankenhausgesellschaft des Landkreis Bamberg Steigerwaldklinik Burgebrach, Burgebrach	Kliniken Nordoberpfalz AG, Krankenhaus Kemnath, Kemnath
GEOMED Klinik Gerolzhofen, Gerolzhofen	Kliniken Nordoberpfalz AG, Krankenhaus Waldsassen, Waldsassen
Goldberg Klinik Kelheim, Kelheim	Kliniken Nordoberpfalz AG, Krankenhaus Tirschenreuth, Tirschenreuth
Haßberg-Kliniken Haus Haßfurt, Haßfurt	Kliniken Ostallgäu Kaufbeuren Klinik St. Josef, Buchloe
Haßberg-Kliniken Haus Ebern, Ebern	Kliniken Ostallgäu Kaufbeuren, Klinik Füssen, Füssen
Helios Amper-Klinik Indersdorf, Indersdorf	Kliniken Südostbayern Kreisklinik Berchtesgaden, Berchtesgaden
Helios Amper-Klinikum Dachau, Dachau	Kliniken Südostbayern Kreisklinik Bad Reichenhall, Bad Reichenhall
HELIOS Frankenwaldklinik Kronach, Kronach	Kliniken Südostbayern Kreisklinik Freilassing, Freilassing
HELIOS Klinik Erlenbach, Erlenbach	Klinikum Altmühlfranken – Gunzenhausen, Gunzenhausen
HELIOS Klinik München Perlach, München	Klinikum Altmühlfranken – Weißenburg, Weißenburg
HELIOS Klinikum München West, München	Klinikum Am Europakanal, Erlangen
HELIOS OrthoCliniC Hammelburg, Hammelburg	Klinikum Aschaffenburg-Alzenau gemeinnützige GmbH - Klinikum Aschaffenburg, Aschaffenburg
HELIOS St. Elisabeth-Krankenhaus Bad Kissingen, Bad Kissingen	Klinikum Aschaffenburg-Alzenau gemeinnützige GmbH - Klinikum Alzenau, Alzenau
Helmut G. Walther Klinikum Lichtenfels, Lichtenfels	Klinikum Bamberg - Klinikum am Bruderwald, Bamberg
Illertalklinik Illertissen, Illertissen	Klinikum Bamberg - Klinikum am Michelsberg und Klinik am Heinrichsdamm, Bamberg
Ilmtalklinik - Krankenhaus Mainburg, Mainburg	Klinikum Bayreuth - Klinik Hohe Warte, Bayreuth
Ilmtalklinik GmbH Pfaffenhofen, Pfaffenhofen	Klinikum Bayreuth, Bayreuth
Internistische Klinik Dr. Steger, München	Klinikum Coburg, Coburg
Internistisches Klinikum München Süd, München	Klinikum der LMU München Innenstadt, München
Isarklinikum, München	Klinikum der LMU München Großhadern, München
kbo-Inn-Salzach-Klinikum Wasserburg, Wasserburg	Klinikum der Universität Erlangen, Erlangen
kbo-Isar-Amper-Klinikum München-Ost, München	Klinikum der Universität Regensburg, Regensburg
Kiliani-Klinik, Bad Windsheim	Klinikum Dritter Orden München – Nymphenburg, München
KJF Klinik Sankt Elisabeth, Neuburg an der Donau	Klinikum Fichtelgebirge gGmbH Haus Marktredwitz, Marktredwitz
Klinik Augustinum München, München	Klinikum Fichtelgebirge gGmbH Haus Selb, Selb
Klinik Bogen, Bogen	Klinikum Forchheim - Fränkische Schweiz gGmbH, Standort Forchheim, Forchheim
Klinik Hallerwiese / Cnopfsche Kinderklinik, Nürnberg	Klinikum Forchheim - Fränkische Schweiz gGmbH, Standort Ebermannstadt, Ebermannstadt
Klinik Kipfenberg, Kipfenberg	Klinikum Freising GmbH, Freising
Klinik Kitzinger Land, Kitzingen	Klinikum Fürstenfeldbruck, Fürstenfeldbruck
Klinik Mallersdorf, Mallersdorf	Klinikum Fürth, Fürth
Klinik Vincentinum Augsburg, Augsburg	Klinikum Garmisch-Partenkirchen Außenstelle Murnau, Murnau
Klinik Wartenberg Prof. Dr. Selmair GmbH & Co. KG, Wartenberg	Klinikum Garmisch-Partenkirchen, Garmisch-Partenkirchen
Kliniken am Goldenen Steig gGmbH Krankenhaus Waldkirchen, Waldkirchen	Klinikum Ingolstadt, Ingolstadt
Kliniken am Goldenen Steig gGmbH Krankenhaus Grafenau, Grafenau	Klinikum Kulmbach, Kulmbach
Kliniken am Goldenen Steig gGmbH Krankenhaus Freyung, Freyung	Klinikum Kulmbach Fachklinik Stadtsteinach, Kulmbach
Kliniken an der Paar Krankenhaus Aichach, Aichach	Klinikum Landkreis Erding Klinikum Erding, Erding
Kliniken an der Paar Krankenhaus Friedberg, Friedberg	Klinikum Landkreis Erding Klinik Dorfen, Dorfen
Kliniken des Landkreises Neumarkt i.d.OPf. Klinikum Neumarkt, Neumarkt	Klinikum Landsberg am Lech, Landsberg am Lech
Kliniken des Landkreises Neumarkt i.d.OPf. Klinik Parsberg, Parsberg	Klinikum Landshut, Landshut
Kliniken des Landkreises Neustadt a. d. Aisch - Bad Windsheim, Bad Windsheim	Klinikum Memmingen, Memmingen
Kliniken des Landkreises Neustadt a. d. Aisch - Klinik Neustadt a. d. Aisch, Neustadt a. d. Aisch	Klinikum Nürnberg Nord, Nürnberg
Kliniken Dr. Erler, Nürnberg	Klinikum Nürnberg Süd, Nürnberg
Kliniken Hochfranken Klinik Münchberg, Münchberg	Klinikum Passau, Passau
Kliniken Hochfranken Klinik Naila, Naila	Klinikum Penzberg, Penzberg
Kliniken im Naturpark Altmühltal Klinik Eichstätt, Eichstätt	
Kliniken im Naturpark Altmühltal Klinik Kösching, Kösching	
Kliniken Kreis Mühldorf a. Inn Klinik Mühldorf, Müldorf	

Supplement Table 1 Name and place of included hospitals that contributed to the quality assurance stroke registries of Northwestern Germany (QSNWD) and of the Bavarian Working Party for Quality Assurance (BAQ) *(continued)*

**BAQ registry**	
Kliniken Kreis Mühldorf a. Inn Klinik Haag, Haag	Klinikum rechts der Isar, München
Kliniken Nordoberpfalz AG, Krankenhaus Neustadt a. d. Waldnaab, Neustadt a. d. Waldnaab	Klinikum Seefeld, Seefeld
Kliniken Nordoberpfalz AG, Klinikum Weiden, Weiden	Klinikum St. Elisabeth Straubing GmbH, Straubing
Kliniken Nordoberpfalz AG, Krankenhaus Vohenstrauß, Vohenstrauß	Klinikum St. Elisabeth Straubing GmbH Akutgeriatrie Bogen, Bogen
Klinikum Starnberg, Starnberg	Klinikum St. Marien Amberg, Amberg
Klinikum Würzburg Mitte gGmbH Juliusspital, Würzburg	LAKUMED Kliniken Schlossklinik Rottenburg, Rottenburg an der Laaber
Klinikum Würzburg Mitte gGmbH Missioklinik, Würzburg	Landkreis Passau Gesundheitseinrichtungen Krankenhaus Wegscheid, Wegscheid
Klinikverbund Kempten-Oberallgäu - Klinik Immenstadt, Immenstadt	Landkreis Passau Gesundheitseinrichtungen Krankenhaus Vilshofen, Vilshofen
Klinikverbund Kempten-Oberallgäu - Klinik Oberstdorf, Oberstdorf	Landkreis Passau Gesundheitseinrichtungen Krankenhaus Rotthalmünster, Rotthalmünster
Klinikverbund Kempten-Oberallgäu - Klinik Sonthofen, Sonthofen	Leopoldina Krankenhaus Schweinfurt, Schweinfurt
Klinikverbund Kempten-Oberallgäu - Klinikum Kempten, Kempten	Main Klinik Ochsenfurt, Ochsenfurt
Klinken Südostbayern Klinikum Traunstein, Traunstein	Malteser Waldkrankenhaus St. Marien, Erlangen
Klinken Südostbayern Kreisklinik Trostberg, Trostberg	Max-Planck-Institut für Psychiatrie, München
Klinken Südostbayern AG Kreisklinik Ruhpolding, Ruhpolding	Medical Park Bad Feilnbach Betriebsstätte Reithofpark, Bad Feilnbach
Krankenhaus Agatharied, Agatharied	München Klinik Bogenhausen, München
Krankenhaus Barmherzige Brüder München, München	München Klinik Harlaching, München
Krankenhaus Barmherzige Brüder Regensburg, Regensburg	München Klinik Neuperlach, München
Krankenhaus für Naturheilweisen, München	München Klinik Schwabing, München
Krankenhaus GmbH Landkreis Weilheim-Schongau Krankenhaus Schongau, Schongau	Privatklinik Dr. Robert Schindlbeck, Herrsching am Ammersee
Krankenhaus GmbH Landkreis Weilheim-Schongau Krankenhaus Weilheim, Weilheim	Regiomed Kliniken - Klinik Neustadt bei Coburg, Neustadt bei Coburg
Krankenhaus Lohr Klinikum Main-Spessart, Lohr	Rhön Klinikum Campus Bad Neustadt a. d. Saale, Bad Neustadt a. d. Saale
Krankenhaus Martha Maria München, München	RoMed Klinik Bad Aibling, Bad Aibling
Krankenhaus Martha Maria Nürnberg, Nürnberg	RoMed Klinik Prien a. Chiemsee, Prien a. Chiemsee
Krankenhaus Neuwittelsbach, Neuwittelsbach	RoMed Klinik Wasserburg, Wasserburg
Krankenhaus Rummelsberg, Rummelsberg	RoMed Klinikum Rosenheim, Rosenheim
Krankenhaus St. Anna, Höchstadt a.d. Aisch	Rotkreuzklinik Lindenberg, Lindenberg
Krankenhaus St. Josef Schweinfurt, Schweinfurt	Rotkreuzklinikum München, München
Krankenhaus Vilsbiburg, Vilsbiburg	Rotkreuzklinikum München Frauenklinik Taxisstraße, München
Krankenhäuser des Landkreises Amberg-Sulzbach - St. Johannes Klinik, Auerbach in der Oberpfalz	Rottal-Inn-Kliniken Eggenfelden, Eggenfelden
Krankenhäuser des Landkreises Amberg-Sulzbach - St. Anna Krankenhaus, Sulzbach-Rosenberg	Rottal-Inn-Kliniken Pfarrkirchen, Pfarrkirchen
Krankenhäuser Nürnberger Land - Krankenhaus Altdorf, Altdorf	Salzachklinik Fridolfing, Fridolfing
Krankenhäuser Nürnberger Land - Krankenhaus Hersbruck, Hersbruck	Sana Klinik Pegnitz, Pegnitz
Krankenhäuser Nürnberger Land - Krankenhaus Lauf, Lauf	Sana Kliniken des Landkreises Cham Krankenhaus St. Josef Bad Kötzting, Bad Kötzting
Kreisklinik Ebersberg, Ebersberg	Sana Kliniken des Landkreises Cham Krankenhaus Roding, Roding
Kreisklinik Roth, Roth	Sana Kliniken des Landkreises Cham Krankenhaus Cham, Cham
Kreisklinik Wolfratshausen, Wolfratshausen	Sana Klinikum Hof, Hof
Kreisklinik Wörth a. d. Donau, Wörth a. d. Donau	Schön Klinik Vogtareuth, Vogtareuth
Kreiskliniken Altötting-Burghausen Kreisklinik Altötting, Altötting	Schön Klinik Bad Aibling, Bad Aibling
Kreiskliniken Altötting-Burghausen, Kreisklinik Burghausen, Burghausen	Schön Klinik Bad Staffelstein, Bad Staffelstein
Kreiskliniken Dillingen – Wertingen Kreiskrankenhaus Wertingen, Wertingen	Schön Klinik Harthausen, Harthausen
Kreiskliniken Dillingen – Wertingen Krankenhaus St. Elisabeth Dillingen, Dillingen	Schön Klinik München Harlaching, München
	Schön Klinik München Schwabing, München
	Spezialklinik Neukirchen, Neukirchen beim Heiligen Blut
	St. Barbara Krankenhaus Schwandorf, Schwandorf

Supplement Table 1 Name and place of included hospitals that contributed to the quality assurance stroke registries of Northwestern Germany (QSNWD) and of the Bavarian Working Party for Quality Assurance (BAQ) *(continued)*

**BAQ registry**	
Kreiskliniken Günzburg-Krumbach Klinik Günzburg, Günzburg	St. Theresien Krankenhaus Nürnberg, Nürnberg
Kreiskliniken Günzburg-Krumbach Klinik Krumbach, Krumbach	St. Vinzenz Klinik Pfronten im Allgäu, Pfronten
Kreiskliniken Unterallgäu Kreisklinik Mindelheim, Mindelheim	Stadtkrankenhaus Schwabach, Schwabach
Kreiskliniken Unterallgäu Kreisklinik Ottobeuren, Ottobeuren	Stiftungsklinik Weißenhorn, Weißenhorn
Kreiskrankenhaus Schrobenhausen GmbH, Schrobenhausen	Therapiezentrum Burgau, Burgau
LAKUMED Kliniken Krankenhaus Landshut – Achdorf, Landshut	Universitätsklinikum Augsburg Medizincampus, Augsburg
	Universitätsklinikum Augsburg Medizincampus Süd, Augsburg
	Universitätsklinikum Würzburg, Würzburg
	Wertachklinik Bobingen, Bobingen
	Wertachklinik Schwabmünchen, Schwabmünchen

Supplement Table 2 Stroke cases in 2017-2020 by quarter in the QSNWD registry

	**2018**				**2019**				**2020**			
	**Q1**	**Q2**	**Q3**	**Q4**	**Q1**	**Q2**	**Q3**	**Q4**	**Q1**	**Q2**	**Q3**	**Q4**
Cases, n	28223	26808	26426	26668	27852	27736	26700	26807	26900	25473	26451	25461
Diagnosis, n (%)												
TIA (ICD-10 G45)	7463 (26.4)	6785 (25.3)	6836 (25.9)	7199 (27.0)	7618 (27.4)	7260 (26.2)	7083 (26.5)	7262 (27.1)	7015 (26.1)	6441 (25.3)	6732 (25.5)	6760 (26.6)
Cerebral infarction (ICD-10 I63)	19048 (67.5)	18598 (69.4)	18213 (68.9)	17876 (67.0)	18667 (67.0)	19025 (68.6)	18203 (68.2)	17931 (66.9)	18330 (68.1)	17533 (68.8)	18288 (69.1)	17215 (67.6)
Cerebral hemorrhage (ICD-10 I61)	1597 (5.7)	1329 (5.0)	1287 (4.9)	1524 (5.7)	1496 (5.4)	1362 (4.9)	1326 (5.0)	1542 (5.8)	1506 (5.6)	1448 (5.7)	1365 (5.2)	1423 (5.6)
Stroke, not specified as hemorrhage or infarction (I64)	115 (0.4)	96 (0.4)	90 (0.3)	69 (0.3)	71 (0.3)	89 (0.3)	88 (0.3)	72 (0.3)	49 (0.2)	51 (0.2)	66 (0.3)	63 (0.3)
NIHSS on admission ≥4 i cases with cerebral infarction, n (%)	10105 (53.2)	9741 (52.6)	9567 (52.7)	9457 (53.0)	9761 (52.4)	9972 (52.5)	9419 (51.9)	9566 (53.5)	9508 (51.9)	8874 (50.7)	9275 (50.8)	8860 (51.6)
Barthel index on admission ≤75, n (%)	13403 (51.7)	12876 (51.8)	12548 (51.4)	12517 (51.0)	12938 (50.3)	12778 (50.1)	12509 (50.5)	12421 (50.5)	12676 (51.0)	11823 (50.3)	12240 (49.9)	11575 (49.4)
Living situation before admission, n (%)												
Independent at home	22444 (79.9)	21499 (80.6)	21300 (80.9)	21390 (80.5)	22323 (80.4)	22223 (80.4)	21442 (80.6)	21582 (80.8)	21490 (80.1)	20567 (81.0)	21177 (80.2)	20425 (80.4)
Care at home	3057 (10.9)	2847 (10.7)	2783 (10.6)	2883 (10.9)	3079 (11.1)	2996 (10.8)	2858 (10.7)	2836 (10.6)	3028 (11.3)	2771 (10.9)	3024 (11.5)	2885 (11.4)
Nursing home	2591 (9.2)	2334 (8.8)	2263 (8.6)	2306 (8.7)	2373 (8.5)	2431 (8.8)	2321 (8.7)	2310 (8.6)	2307 (8.6)	2062 (8.1)	2191 (8.3)	2104 (8.3)
Duration of stay in hospital in full days, median (IQR)	7 (4-11)	7 (4-11)	6 (4-10)	7 (4-11)	7 (4-11)	7 (4-11)	6 (4-10)	7 (4-11)	6 (4-10)	6 (4-10)	6 (4-10)	6 (4-10)
Duration of stay on stroke unit in full days, median (IQR)	3 (1-4)	3 (1-4)	3 (1-4)	3 (1-4)	3 (1-3)	3 (1-3)	3 (1-3)	3 (1-4)	3 (1-3)	3 (1-4)	3 (1-4)	2 (1-3)

IQR, interquartile range; NIHSS, National Institutes of Health Stroke Scale; TIA, transient ischemic attack.

Supplement Table 2 Stroke cases in 2017-2020 by quarter in the QSNWD registry *(continued)*

	2018				2019				2020			
	**Q1**	**Q2**	**Q3**	**Q4**	**Q1**	**Q2**	**Q3**	**Q4**	**Q1**	**Q2**	**Q3**	**Q4**
Female sex, n (%)	13606 (48.2)	12794 (47.8)	12657 (47.9)	12894 (48.4)	13317 (47.9)	13316 (48.0)	12844 (48.1)	13084 (48.8)	12903 (48.0)	12060 (47.4)	12682 (48.0)	12155 (47.8)
Age, median (IQR)	76 (64-82)	76 (64-82)	76 (64-82)	76 (65-83)	76 (65-83)	76 (65-83)	76 (64-83)	76 (65-83)	76 (64-83)	76 (65-83)	76 (64-83)	76 (65-83)
Time interval event to admission, n (%)												
≤1h	2099 (7.4)	2000 (7.5)	2013 (7.6)	2089 (7.8)	2030 (7.3)	2098 (7.6)	1941 (7.3)	2036 (7.6)	2033 (7.6)	1746 (6.9)	1823 (6.9)	1688 (6.6)
>1-2h	4756 (16.9)	4407 (16.4)	4320 (16.4)	4533 (17.0)	4559 (16.4)	4537 (16.4)	4122 (15.4)	4506 (16.8)	4501 (16.7)	4357 (17.1)	4256 (16.1)	4228 (16.6)
>2-3h	3116 (11.0)	3020 (11.3)	2916 (11.0)	2909 (11.0)	3021 (10.9)	3131 (11.3)	2956 (11.1)	2990 (11.2)	2982 (11.1)	2751 (10.8)	2862 (10.8)	2951 (11.6)
>3-4h	1945 (6.9)	1856 (6.9)	1825 (6.9)	1867 (7.0)	1981 (7.1)	1950 (7.0)	1907 (7.1)	1980 (7.4)	1758 (6.5)	1702 (6.7)	1812 (6.9)	1811 (7.1)
>4-5h	1431 (5.1)	1348 (5.0)	1259 (4.8)	1314 (4.9)	1397 (5.0)	1314 (4.7)	1358 (5.1)	1395 (5.2)	1344 (5.0)	1188 (4.7)	1297 (4.9)	1178 (4.6)
>5-6h	1264 (4.5)	1182 (4.4)	1258 (4.8)	1283 (4.8)	1385 (5.0)	1342 (4.8)	1336 (5.0)	1268 (4.7)	1314 (4.9)	1299 (5.1)	1279 (4.8)	1193 (4.7)
>6-24h	6098 (21.6)	5798 (21.6)	5463 (20.7)	5677 (21.3)	6293 (22.6)	6165 (22.2)	6090 (22.8)	5949 (22.2)	6224 (23.1)	6016 (23.6)	6100 (23.1)	6231 (24.5)
>24-48h	2052 (7.3)	1991 (7.4)	1985 (7.5)	1779 (6.7)	1883 (6.8)	2001 (7.2)	1882 (7.1)	1815 (6.8)	1870 (7.0)	1780 (7.0)	1902 (7.2)	1647 (6.5)
>48h-7 days	2787 (9.9)	2802 (10.5)	2870 (10.9)	2590 (9.7)	2750 (9.9)	2763 (10.0)	2753 (10.3)	2481 (9.3)	2560 (9.5)	2551 (10.0)	2841 (10.7)	2366 (9.3)
In-house stroke	265 (0.9)	247 (0.9)	201 (0.8)	238 (0.9)	301 (1.1)	275 (1.0)	296 (1.1)	287 (1.1)	215 (0.8)	198 (0.8)	180 (0.7)	194 (0.8)
Unknown	2410 (8.5)	2157 (8.1)	2316 (8.8)	2389 (9.0)	2252 (8.1)	2160 (7.8)	2059 (7.7)	2100 (7.8)	2099 (7.8)	1885 (7.4)	2099 (7.9)	1974 (7.8)

IQR, interquartile range.

Supplement Table 3 Stroke cases in 2017-2020 by quarter in the BAQ registry

	2018				2019				2020			
	Q1	Q2	Q3	Q4	Q1	Q2	Q3	Q4	Q1	Q2	Q3	Q4
Cases, n	12969	12479	12155	12291	12632	12396	12381	12826	12305	11174	11879	10939
Diagnosis, n (%)												
TIA (ICD-10 G45)	3598 (27.7)	3398 (27.2)	3359 (27.6)	3323 (27.0)	3577 (28.3)	3552 (28.7)	3469 (28.0)	3692 (28.8)	3335 (27.1)	2985 (26.7)	3127 (26.3)	3033 (27.7)
Cerebral infarction (ICD-10 I63)	8544 (65.9)	8280 (66.4)	8039 (66.1)	8129 (66.1)	8173 (64.7)	8083 (65.2)	8102 (65.4)	8295 (64.7)	8164 (66.4)	7425 (66.5)	7958 (67.0)	7126 (65.1)
Cerebral hemorrhage (ICD-10 I61)	755 (5.8)	720 (5.8)	682 (5.61)	751 (6.1)	806 (6.4)	700 (5.7)	714 (5.8)	788 (6.1)	751 (6.1)	721 (6.5)	717 (6.0)	676 (6.2)
Stroke, not specified as hemorrhage or infarction (I64)	72 (0.6)	81 (0.7)	75 (0.6)	88 (0.7)	76 (0.6)	61 (0.5)	96 (0.8)	51 (0.4)	55 (0.5)	43 (0.4)	77 (0.7)	104 (1.0)
NIHSS on admission ≥4 in cases with cerebral infarction, n (%)	4220 (49.5)	3959 (47.9)	3875 (48.4)	3967 (48.9)	3939 (48.3)	3821 (47.4)	3786 (46.9)	4031 (48.8)	3949 (48.6)	3503 (47.4)	3751 (47.4)	3400 (48.0)
Barthel index on admission ≤75, n (%)	4840 (38.5)	4425 (36.5)	4352 (36.8)	4581 (38.3)	4773 (38.7)	4463 (36.9)	4350 (36.1)	4743 (38.0)	4674 (38.8)	4165 (38.1)	4318 (37.2)	3847 (36.2)
Living situation before admission, n (%)												
Independent at home	10354 (79.8)	10104 (81.0)	9778 (80.4)	9860 (80.2)	10032 (79.4)	9961 (80.4)	10006 (80.8)	10224 (79.7)	9760 (79.3)	9047 (81.0)	9534 (80.3)	8868 (81.1)
Care at home	1436 (11.1)	1324 (10.6)	1274 (10.5)	1260 (10.3)	1436 (11.4)	1349 (10.9)	1319 (10.7)	1458 (11.4)	1436 (11.7)	1275 (11.4)	1296 (10.9)	1194 (10.9)
Nursing home	1179 (9.1)	1051 (8.4)	1103 (9.1)	1171 (9.5)	1164 (9.2)	1086 (8.8)	1056 (8.5)	1144 (8.9)	1109 (9.0)	852 (7.6)	1049 (8.8)	877 (8.0)
Duration of stay in hospital in full days, median (IQR)	6 (4-10)	6 (3-9)	6 (4-9)	6 (4-9)	6 (3-9)	6 (3-9)	6 (3-9)	6 (3-9)	5 (3-9)	6 (3-9)	5 (3-9)	5 (3-8)
Duration of stay on stroke unit in full days, median (IQR)	NR	NR	NR	NR	NR	NR	NR	NR	NR	NR	NR	NR

IQR, interquartile range; NIHSS, National Institutes of Health Stroke Scale; NR, not reported; TIA, transient ischemic attack.

Supplement Table 3 Stroke cases in 2017-2020 by quarter in the BAQ registry *(continued)*

	2018				2019				2020			
	**Q1**	**Q2**	**Q3**	**Q4**	**Q1**	**Q2**	**Q3**	**Q4**	**Q1**	**Q2**	**Q3**	**Q4**
Female sex, n (%)	6312 (48.7)	5982 (47.9)	5888 (48.4)	6014 (48.9)	6180 (48.9)	5871 (47.4)	5922 (47.8)	6305 (49.2)	5982 (48.7)	5220 (46.8)	5693 (47.9)	5203 (47.6)
Age, median (IQR)	77 (67-83)	77 (65-83)	77 (66-8)	77 (66-83)	77 (66-83)	77 (66-83)	77 (65-83)	77 (66-83)	77 (66-83)	77 (66-83)	77 (66-83)	77 (66-83)
Time interval event to admission, n (%)												
≤1h	1037 (8.0)	974 (7.8)	924 (7.6)	1035 (8.4)	937 (7.4)	1024 (8.3)	942 (7.6)	1053 (8.2)	1075 (8.7)	897 (8.0)	992 (8.4)	915 (8.4)
>1-2h	2334 (18.0)	2175 (17.4)	2040 (16.8)	2192 (17.8)	2091 (16.6)	2077 (16.8)	2042 (16.5)	2174 (17.0)	2179 (17.7)	2062 (18.5)	2024 (17.0)	1847 (16.9)
>2-3h	1412 (10.9)	1302 (10.4)	1426 (11.7)	1365 (11.1)	1405 (11.1)	1338 (10.8)	1239 (10.0)	1375 (10.7)	1259 (10.2)	1152 (10.3)	1272 (10.7)	1205 (11.0)
>3-4h	910 (7.0)	843 (6.8)	848 (7.0)	839 (6.8)	915 (7.2)	824 (6.7)	872 (7.0)	927 (7.2)	799 (6.5)	713 (6.4)	819 (6.9)	732 (6.7)
>4-5h	649 (5.0)	615 (4.9)	610 (5.0)	548 (4.5)	634 (5.0)	582 (4.7)	563 (4.6)	611 (4.8)	545 (4.4)	565 (5.1)	505 (4.3)	508 (4.6)
>5-6h	542 (4.2)	531 (4.3)	488 (4.0)	562 (4.6)	523 (4.1)	570 (4.6)	575 (4.6)	538 (4.2)	452 (3.7)	425 (3.8)	481 (4.1)	415 (3.8)
>6-24h	2771 (21.4)	2549 (20.4)	2658 (21.9)	2586 (21.0)	2924 (23.2)	2950 (23.8)	3067 (24.8)	3069 (23.9)	3031 (24.6)	2657 (23.8)	2812 (23.7)	2720 (24.9)
>24-48h	900 (6.9)	983 (7.9)	872 (7.2)	820 (6.7)	874 (6.9)	811 (6.5)	913 (7.4)	869 (6.8)	825 (6.7)	775 (6.9)	805 (6.8)	740 (6.8)
>48h – 7 days	1125 (8.7)	1197 (9.6)	1142 (9.4)	1117 (9.1)	1203 (9.5)	1231 (9.9)	1178 (9.5)	1137 (8.9)	1075 (8.7)	1042 (9.3)	1096 (9.2)	979 (9.0)
In-house stroke	110 (0.9)	108 (0.9)	92 (0.8)	89 (0.7)	114 (0.9)	98 (0.8)	87 (0.7)	94 (0.7)	76 (0.6)	63 (0.6)	64 (0.5)	77 (0.7)
unknown	1179 (9.1)	1202 (9.6)	1055 (8.7)	1138 (9.3)	1012 (8.0)	891 (7.2)	903 (7.3)	979 (7.6)	989 (8.0)	823 (7.4)	1009 (8.5)	801 (7.3)

IQR, interquartile range.

Supplement Table 4 Chi-squared tests for changes in the registries QSNWD and BAQ from Q2 and Q4 2019 to Q2 and Q4 2020, respectively

	**Q2 2019**	**Q2 2020**	**Difference Q2 2020 - Q2 2019**	**p-Value of chi-square test**	**Q4 2019**	**Q4 2020**	**Difference Q4 2020 - Q4 2019**	**p-Value of chi-square test**
Systemic thrombolysis								
QSNWD	69.22	70.98	1.76	0.222	66.73	70.97	4.24	0.004*
BAQ	70.49	73.86	3.37	0.132	72.85	73.86	1.01	0.659
Intra-arterial therapy								
QSNWD	72.19	73.78	1.59	0.343	73.01	76.81	3.8	0.022*
BAQ	78.88	79.93	1.05	0.676	78.34	73.79	-4.55	0.091
Death in hospital in cases with cerebral infarction								
QSNWD	6.25	6.25	0	0.981	6.54	6.49	-0.05	0.981
BAQ	5.58	6.49	0.91	0.017*	6.44	7.16	0.72	0.076
Complication bleeding								
QSNWD	1.25	1.07	-0.18	0.045*	1.18	1.1	-0.08	0.395
BAQ	0.95	1.29	0.34	0.014*	1.26	1.12	-0.14	0.355
Time between admission and thrombolysis ≤60 minutes								
QSNWD	84.72	84.33	-0.39	0.777	83.68	85.29	1.61	0.249
BAQ	86.56	87.68	1.12	0.567	87.81	86.64	-1.17	0.554
Pneumonia								
QSNWD	5.08	5.15	0.07	0.713	4.99	5.31	0.32	0.091
BAQ	3.65	4.19	0.54	0.035*	4.37	4.35	-0.02	0.956
NIHSS on admission ≥4 in cases with cerebral infarction								
QSNWD	52.54	50.72	-1.82	0.001*	53.48	51.57	-1.91	<0.001*
BAQ	47.39	47.43	0.04	0.956	48.76	47.98	-0.78	0.336
Barthel index on admission ≤75								
QSNWD	50.05	50.31	0.26	0.569	50.54	49.36	-1.18	0.009*
BAQ	36.9	38.1	1.23	0.054	38.00	36.19	-1.81	0.005*
Sex								
QSNWD	48.04	47.38	-0.66	0.123	48.84	47.77	-1.07	0.015*
BAQ	47.37	46.76	-0.61	0.354	49.17	47.57	-1.6	0.014*

* P-values of chi-squared tests < 0.05

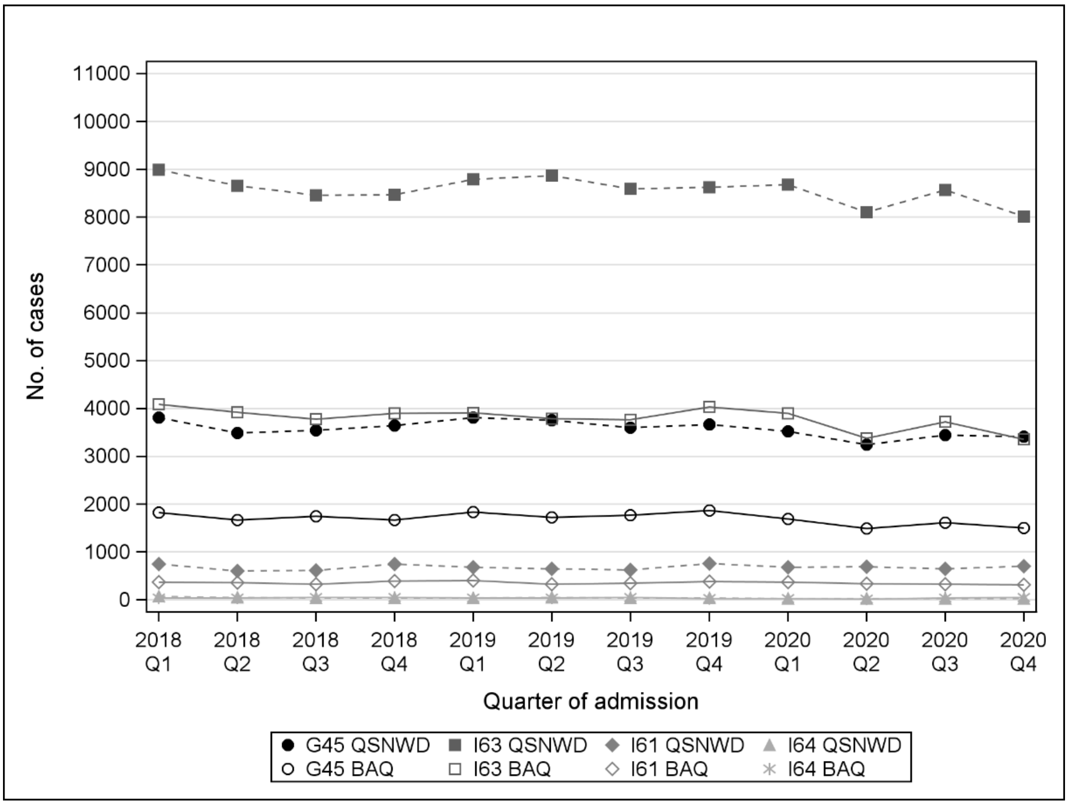

Supplement Figure 1 Number of women with transient ischemic attack (ICD-10 G45), cerebral infarction (ICD-10 I63), cerebral hemorrhage (ICD-10 I61) or stroke, not specified as hemorrhage or infarction (I64) from the registries QSNWD (dashed lines) and BAQ (solid lines) in 2018-2020 by quarter of admission

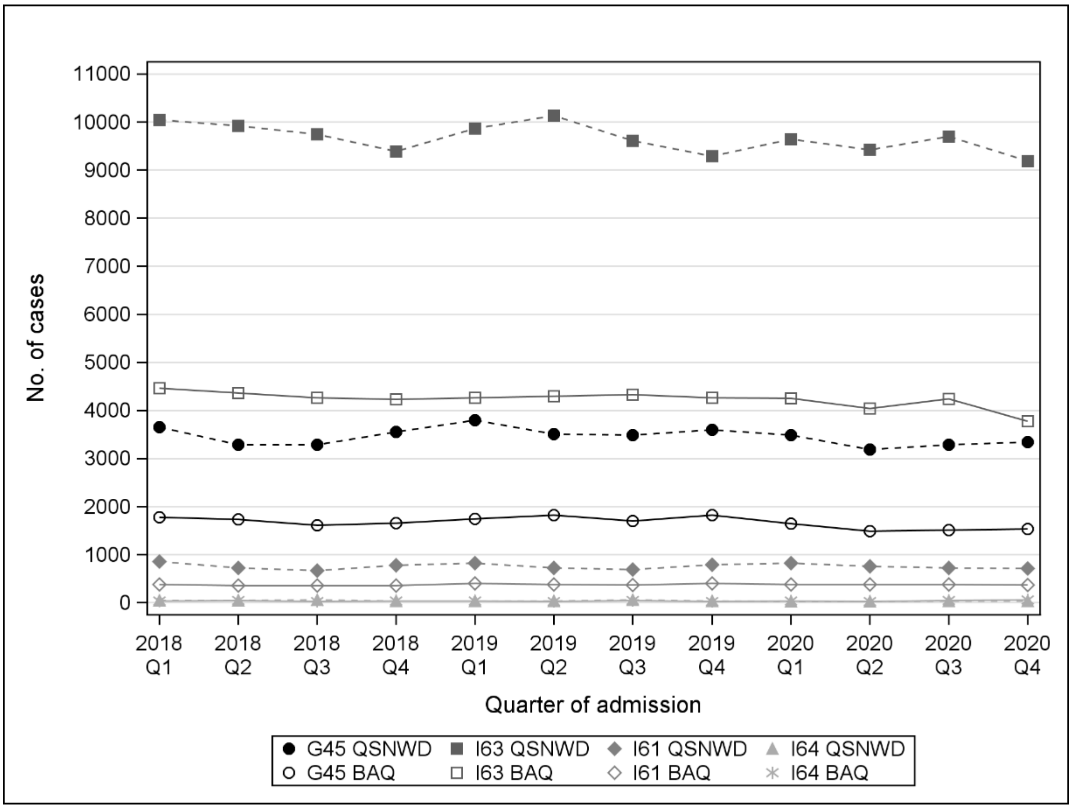

Supplement Figure 2 Number of men with transient ischemic attack (ICD-10 G45), cerebral infarction (ICD-10 I63), cerebral hemorrhage (ICD-10 I61) or stroke, not specified as hemorrhage or infarction (I64) from the registries QSNWD (dashed lines) and BAQ (solid lines) in 2018-2020 by quarter of admission

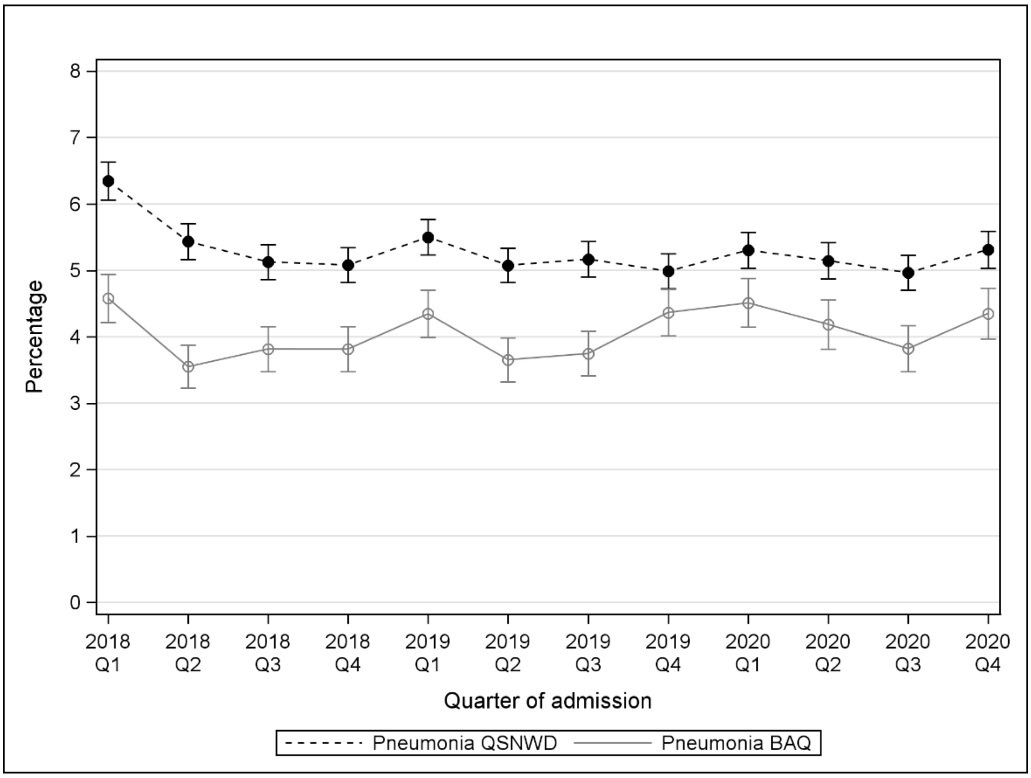

Supplement Figure 3 Pneumonia of patients from the registries QSNWD (dashed line) and BAQ (solid line) in 2018-2020 by quarter of admission
